# Performance of risk prediction scores for cardiovascular mortality in older persons: External validation of the SCORE OP and appraisal

**DOI:** 10.1371/journal.pone.0231097

**Published:** 2020-04-09

**Authors:** Marco Piccininni, Jessica L. Rohmann, Dörte Huscher, Nina Mielke, Natalie Ebert, Giancarlo Logroscino, Elke Schäffner, Tobias Kurth

**Affiliations:** 1 Institute of Public Health, Charité –Universitätsmedizin Berlin, Berlin, Germany; 2 Department of Basic Medical Sciences, Neuroscience and Sense Organs, University of Bari Aldo Moro, Bari, Italy; 3 Center for Neurodegenerative Diseases and the Aging Brain, Department of Clinical Research in Neurology, University of Bari Aldo Moro, Pia Fondazione Cardinale G Panico, Tricase, Italy; 4 Institute of Biometry and Clinical Epidemiology, Charité –Universitätsmedizin Berlin, Berlin, Germany; Icahn School of Medicine at Mount Sinai, UNITED STATES

## Abstract

**Background:**

European guidelines recommend the use of the Systematic COronary Risk Evaluation (SCORE) to assess 10-year risk of fatal cardiovascular events in people aged 40 to 65. The SCORE Older Persons (SCORE OP, 5-year and 10-year versions) was recently developed for people aged 65 or older. We assessed the performance of these risk scores in predicting fatal cardiovascular events in older persons in Berlin.

**Methods and findings:**

Data from the Berlin Initiative Study (BIS), a prospective, population-based study of older persons recruited from a German public health insurance company database were used. 1,657 participants aged 70 or older without reported previous myocardial infarction were included. We assessed calibration by comparing predicted risks to observed (for 5-year versions, 5y) or projected (for 10-year versions) probabilities. During follow-up (median: 4.8 years), 118 cardiovascular deaths occurred. The calibration assessment of the SCORE OP-H 5y and SCORE OP-L 5y equations revealed 2.1- and 1.5-fold overestimation. Comparing 10-year versions, the SCORE OP showed better discrimination ability compared to the SCORE (C-indices of around 0.80 compared to 0.72) and the SCORE for high-risk regions showed the best calibration (chi-square = 29.68). The SCORE OP overestimated the true risk; 519 and 677 events were predicted using the low-risk and high-risk region SCORE OP equations compared to 397 to 399 events projected based on BIS follow-up data (predicted/actual ratios of 1.3 and 1.7).

**Conclusions:**

Given the low transportability of the SCORE OP observed in our population, we caution against its use in routine clinical practice until further information is available to avoid possible overtreatment among older persons in Berlin.

## Introduction

Cardiovascular (CV) diseases are a leading cause of morbidity and are responsible for approximately 40% of deaths in the European Union[1] and 30% of deaths worldwide[2]. As most major CV risk factors are modifiable and can be targeted in preventive strategies[3–6], application of risk stratification tools remain of increasing importance and is suggested in the majority of CV disease guidelines[7,8].

More than 363 prediction models for CV disease have been developed over the last decades[9]. Generally, most known are the risk scores developed in the Framingham Heart Study[10–12]. The Systematic COronary Risk Evaluation (SCORE) was developed in 2003 to predict the 10-year risk of CV disease mortality in European populations using data from twelve European cohort studies[13]. The original SCORE is recommended for individuals aged 40 to 65 by the European guidelines on CV disease prevention from the European Society of Cardiology[14], and has been re-calibrated in various European countries to account for differences in mortality rates and risk factor distributions[15–18].

Since the majority of risk scores have been developed using data from primarily middle-aged populations, it remains unknown how well they perform in older populations[19,20]. With steadily increasing life expectancy, the need for valid CV risk assessment tools for older individuals is becoming more pressing[21,22]. Indeed, fewer than 20% of all fatal CV events occur between ages 40 and 65 in Europe[23]. Furthermore, since relationships between individual risk factors and CV events are known to change with age[19], it seems unlikely that fatal CV event risk scores based on coefficients estimated from a mostly middle-aged population provide reliable estimates of the actual probabilities in older persons[20,22].

Based on this rationale, an updated version of SCORE was developed in 2015 to predict the risk of fatal CV events specifically for persons aged ≥65 (SCORE Older Persons, SCORE OP)[22].

Until recently, the SCORE OP had not been externally validated; the newly published study calls for further validations of this risk tool[24]. Our objective was to assess calibration and discrimination of SCORE OP in a prospective cohort study of individuals aged ≥70 in Berlin. Our secondary aim was to compare the 10-year predictive performance of SCORE OP and SCORE in this cohort.

## Methods

### Study population

We used data from the ongoing Berlin Initiative Study, a longitudinal, population-based cohort study of adults aged ≥70 with biennial follow-up visits. Details of the study design have been previously described[25]. In brief, starting in November 2009, participants were selected using age and sex-stratified random sampling from a database of one of the largest German health insurance companies in the Berlin region (Allgemeine Ortskrankenkasse (AOK) Nordost) that covers about 50% of older persons in the Berlin region. Oversampling was conducted to increase participation among women and the highest age strata[26]. At the end of the recruitment in June 2011, a total of 2,069 individuals (52.6% female, mean age 80.4 years) completed baseline assessment at one of 13 clinical centers across Berlin. The response rate of the random sample was 8.1%, and the distribution of common comorbidities, including myocardial infarction and stroke, was found to be representative of the Allgemeine Ortskrankenkasse Nordost source population[26]. An administrative censorship date of September 30th, 2015 was used to ensure endpoint information completeness. In accordance with the eligibility criteria used to select participants in the development of the original SCORE risk scores, all participants with follow-up information who self-reported no previous history of myocardial infarction at baseline were included. Written informed consent was obtained from all participants prior to recruitment. The Berlin Initiative Study was approved by the ethics committee of Charité –Universitätsmedizin Berlin, Germany (EA2/009/08).

### Risk scores

The following risk scores were selected for external validation:

- SCORE OP-H 5y: European risk score for 5-year fatal CV events among older persons in high-risk regions[22]
- SCORE OP-L 5y: European risk score for 5-year fatal CV events among older persons in low-risk regions[22]
- SCORE OP-H: European risk score for 10-year fatal CV events among older persons in high-risk regions[22]
- SCORE OP-L: European risk score for 10-year fatal CV events among older persons in low-risk regions[22]
- SCORE-H: European risk score for 10-year fatal CV events among adults in high-risk regions[13]
- SCORE-L: European risk score for 10-year fatal CV events among adults in low-risk regions[13]

Since Germany is considered to be between a high- and low-risk region[27], we validated both versions (for definitions, see [14]).

The published formulas, parameters, and coefficients were used for the computation of the risk scores. Of the two original parallel SCORE estimation models, we used the simpler version (with total cholesterol), since no superiority was demonstrated by the model including total cholesterol to the high-density lipoprotein cholesterol ratio.[13] Additionally, we used the corrected version of the original SCORE formula, which was characterized by a mathematical inconsistency.[28] In the 2003 publication, the overall risk for fatal CV events (***R***) was estimated as the sum of two risks; the risk for coronary heart disease death (***RCHD***) and risk for non-coronary CV disease death (***Rnon-CHD CVD***) as follows:

$$R=R_{CHD}+R_{non-CHD\;CVD}$$

Defining the risk of CV mortality in this way is not suitable for participants with high-risk profiles since the sum of the two specific risks can exceed one. For this reason, we used a simple correction to estimate the overall risk, assuming cause-specific risk independence:

$$R=1–\left(1–R_{CHD}\right)\cdot \left(1–R_{non-CHD\;CVD}\right)$$

This correction was initially proposed by Støvring *et al*. as the first step in their approach aiming to account for competing events in the SCORE model.[28]

Since we are validating these scores in an increased risk population due to advanced age, we have applied this approach to avoid implausible probabilities exceeding 100%.

### Risk factors

The following variables used in the models were assessed at baseline during a face-to-face interview: age as an integer, sex, the mean value of two consecutive blood pressure measurements, HDL and total cholesterol levels (converted from mg/dl to mmol/l dividing by 38.67), self-reported current smoking status, diabetes mellitus status (as determined by self-reported use of antidiabetic medication and/or measured HbA1c level >6.5%), and self-reported use of antihypertensive medication.

### Endpoint definition

In the Berlin Initiative Study, information about participant deaths was obtained from several sources. When no reply to a follow-up invitation letter was received and the participant could not be reached, the general practitioner on file for the participant was contacted to inquire about vital status (and date of death, if deceased). Occasionally, participants’ relatives contacted the study team directly about death events. If no information could be obtained from these sources, AOK Nordost records were used to determine vital status. Berlin Initiative Study staff additionally obtained records from the Berlin death certificates archive to validate all deaths, confirm dates, and obtain information on causes of death. Independent of the death certificate information, the study team attempted to obtain archived medical discharge letters for all in-hospital deaths as supplemental information to help correctly classify the cause of death. CV death was defined as death due to fatal myocardial infarction, fatal coronary heart disease, sudden cardiac death, death due to other cardiac diseases such as heart failure, fatal cerebrovascular disease (ischemic stroke, subarachnoid hemorrhage, intracerebral hemorrhage), and death due to peripheral occlusive arterial disease (complications of an aortic aneurysm, organ ischemia, ischemia). Ambiguous cause of death information were discussed and final coding decisions were made by two medical doctors without knowledge of individuals’ risk factor profiles. In cases where mortality was confirmed but cause of death could not be ascertained due to insufficient information, we assumed death by non-CV cause in the primary analysis.

### Statistical analyses

Participants’ baseline risk factors are reported as frequencies and percentages or using means and standard deviations for categorical and continuous variables. Person-years were calculated from the date of recruitment to the individual’s death date (as confirmed by a death certificate), or the date of the last available study visit in cases of loss to follow-up, or the date of administrative censorship (September 30, 2015).

Associations between the predicted risks from each of the risk estimation systems were assessed using pairwise Spearman's rank correlation coefficients. We then computed overall and CV-specific mortality rates as ratios of the number of observed events to the total amount of observed person-years with 95% Poisson exact confidence intervals.

For each prognostic model, and for both high- and low-risk region versions, we computed predicted risks for all individuals. In a second step, we grouped individuals according to deciles of the predicted risk and assessed calibration for each predicted risk decile group. The “actual” probability of an event was compared to the mean of the predicted risks in each decile group. This comparison was conducted both graphically using a calibration plot and, to allow for comparability with previous studies, using the Nam-D’Agostino chi-square test[29]. We further compared the overall number of “actual” and predicted events across the decile groups to assess calibration-in-the-large for each risk score.

Given that the Berlin Initiative Study follow-up was shorter than 10 years, we performed calibration assessment using five years of the observed follow-up data for the SCORE OP risk equations (SCORE OP-H 5y and SCORE OP-L 5y) as the primary analysis. In this analysis, the calibration assessment was performed using the Kaplan-Meier estimates as “actual” probabilities. The original SCORE-L and SCORE-H risk equations were unfortunately only reported as 10-year versions. For calibration assessment of the SCORE risk equations, it was therefore necessary to project probabilities of the endpoints beyond the observed follow-up. Specifically, after creating the predicted risk decile groups, we ran Weibull regression survival models treating the predicted risk groups as a categorical covariate and the 10-year probabilities for each decile group projected by the Weibull models were considered “actual” probabilities. In this secondary analysis, assessment of 10-year calibration was also performed for the SCORE OP risk equations.

The discrimination ability of each risk score was assessed using the concordance index (C-index)[30] on the entire observed follow-up.

We computed projected probability estimates and the C-index along with their 95% bias-corrected and accelerated bootstrapped confidence intervals with 2,000 bootstrap replications.

To check for robustness of our primary results treating unknown causes of death as non-CV, we re-ran all analyses making the most extreme assumption that all deaths of unknown cause were of CV nature. Moreover, to confirm that our external validation study findings are applicable to the general older Berlin population as a whole, we performed an additional sensitivity analysis. All analytical steps described above were repeated in five resampled datasets (by age and sex strata of the BIS study population), which were generated to create a pseudopopulation exactly representative in terms of size and demographic structure of the 2010 Berlin population aged 70 or older (data from [31]; see S1 File).

To explore the possible impact on clinical decision making, we applied different “very-high-risk” thresholds, above which prevention and/or treatment intervention strategies would be indicated among older persons. Since no such single very-high-risk threshold has been established, we compared five different hypothetical thresholds (≥10%, ≥15%, ≥20%, ≥25%, and ≥30%) and calculated the number of very-high-risk persons based on 10-year predicted risks using the various risk scores. We also computed the percentage of participants classified as very-high-risk at the various thresholds based on a full Weibull model (with sex, systolic blood pressure, total cholesterol, HDL cholesterol, smoking status, diabetes and age as covariates and cardiovascular fatal events as the outcome) fitted on BIS data to give a near description of the projected reality at 10 years for our cohort.

All statistical analyses were performed using R v3.4.3 (https://www.R-project.org/) and RStudio v1.0.153 (https://www.rstudio.com/).

## Results

**Fig 1:**
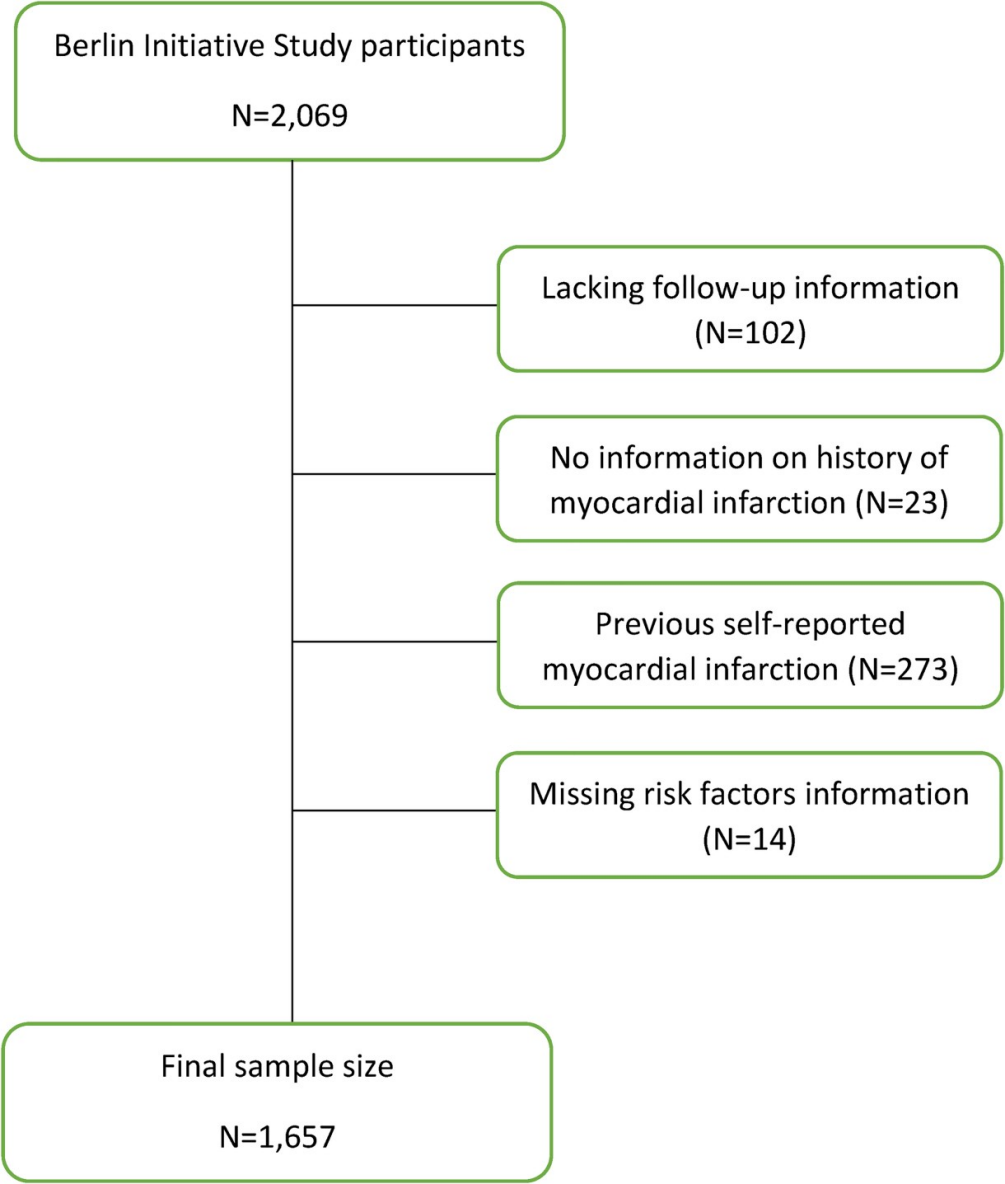
Flow chart showing berlin initiative study participant inclusion/exclusion criteria for this external validation study.

Of the original 2,069 Berlin Initiative Study participants, 412 people who self-reported a history of myocardial infarction or had missing information on past myocardial infarction, on one or more risk factors included in the SCORE OP equations, or lacked any follow-up were excluded (Fig 1). Baseline characteristics of the 1,657 remaining included participants, as well as mean values for predicted risks estimated by SCORE OP-H 5y, SCORE OP-L 5y, SCORE-H, SCORE-L, SCORE OP-H, and SCORE OP-L are displayed in Table 1. During the observed follow-up period (median: 4.8 years), a total of 324 deaths were recorded, of which 118 (36.4%) were CV deaths. In total, participants contributed 7,370.3 person-years, and the overall mortality and CV-specific fatal event rates in the cohort were 44.0 (39.3 to 49.0) and 16.0 (13.3 to 19.2) per 1,000 person-years.

**Table 1:** Baseline characteristics of the study population^a^.

	Total (n = 1,657)		Males (n = 734)		Females (n = 923)	
**Mean age (SD), years**	79.7	(6.7)	80.2	(6.7)	79.2	(6.6)
**Current smoking, N (%)**	86	(5.2%)	47	(6.4%)	39	(4.2%)
**Diabetes, N (%)**	414	(25.0%)	200	(27.2%)	214	(23.2%)
**Hypertensive treatment, N (%)**	1254	(75.9%)	551	(75.3%)	703	(76.3%)
**Cholesterol (SD), mmol/l**	5.6	(1.2)	5.1	(1.1)	5.9	(1.2)
**HDL cholesterol (SD), mmol/l**	1.5	(0.5)	1.3	(0.4)	1.7	(0.4)
**Mean systolic blood pressure, mmHg (SD)**	147.0	(22.8)	147.3	(22.8)	146.7	(22.7)
**Mean diastolic blood pressure, mmHg (SD)**	82.0	(14.5)	82.4	(14.6)	81.6	(14.4)
**Risk scores**^**b**^						
SCORE OP-H 5y risk (SD)	0.18	(0.17)	0.20	(0.16)	0.17	(0.17)
SCORE OP-L 5y risk (SD)	0.13	(0.13)	0.16	(0.14)	0.10	(0.12)
SCORE-H risk (SD)	0.22	(0.15)	0.27	(0.15)	0.19	(0.13)
SCORE-L risk (SD)	0.16	(0.11)	0.17	(0.11)	0.14	(0.11)
SCORE OP-H risk (SD)	0.41	(0.27)	0.45	(0.25)	0.37	(0.27)
SCORE OP-L risk (SD)	0.31	(0.24)	0.36	(0.23)	0.28	(0.24)

^a^All participants were enrolled between 2009–2011.

^b^SCORE OP[22] and SCORE[13] risk scores are described in detail in the Methods section. H and L indicate high- and low- cardiovascular risk regions. Unless otherwise specified, 10-year risk equations were used. 5y indicates 5-year risk equations.

A correlation plot illustrates the distribution of the participants’ predicted probabilities of the various prognostic models and correlations between the individual scores (S1 Fig in S1 File). As expected, both high-risk and low-risk region pairs from the same models were highly correlated (Spearman’s rho 0.99–1.00), as well as the 5- and 10-year versions of the SCORE OP (rho = 0.99–1.00). Overall, we observed moderate correlation between all SCORE and SCORE OP equations (0.76–0.78).

**Fig 2:**
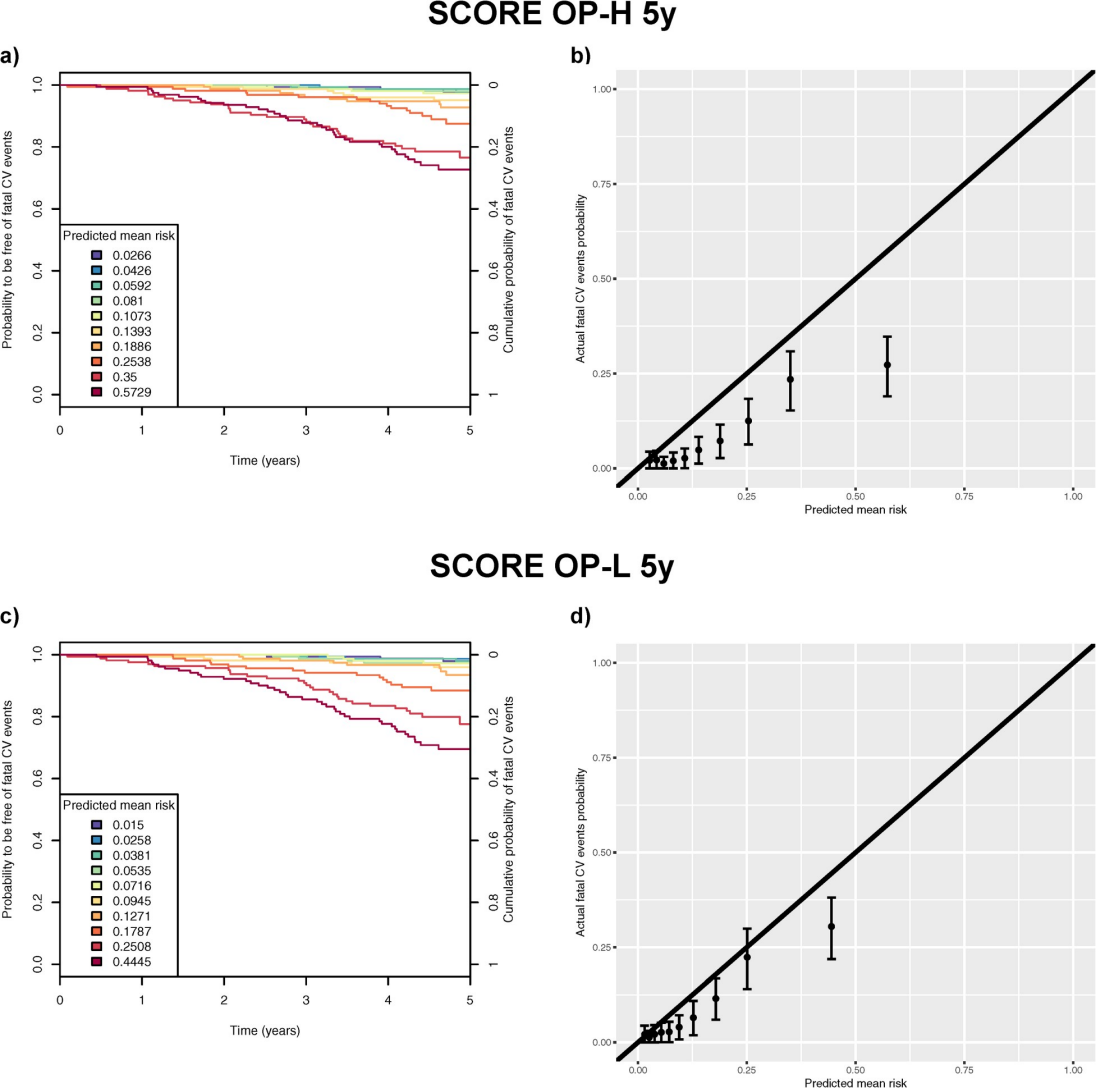
Panel a) shows observed Kaplan-Meier probabilities to be free of fatal cardiovascular (CV) events at a given time point grouped by deciles of risk as predicted by SCORE OP-H 5y (5-year risk equation for high-risk regions). The right y-axis scale shows the probability of the complementary event: occurrence of a fatal CV event before a given time point in the counterfactual scenario of no competing events (if we assume independent competing risks). The legend indicates the average predicted risk of having a fatal CV event within each decile group of risk. Panel b) shows the calibration plot for SCORE OP-H 5y comparing the predicted mean risk (corresponding to ones in the legend of Panel a)) to the actual fatal CV event probabilities within five years (corresponding to the intersection between the curves and the right Y-axis in Panel a)) for each decile group. We report 95% confidence intervals. Panels c) and d) show the results as described in Panels a) and b) for SCORE OP-L 5y (5-year risk equation for low-risk regions).

The calibration of both the SCORE OP-H 5y and the SCORE OP-L 5y equations were assessed using observed probabilities (Fig 2). In total, 302 fatal CV events were predicted by the SCORE OP-H 5y while 142 fatal CV events were estimated without accounting for competing risks, showing an overestimation (predicted/actual ratio = 2.13). This score systematically overestimated the true risk (chi-square = 139.2, Fig 2A and 2B). The SCORE OP-L 5y also showed overestimation, but to a lesser extent, with 215 predicted compared to 142 observed events (ratio = 1.51, chi-square = 39.7, Fig 2C and 2D). Again, a systematic overestimation was observed across most of the risk score decile groups.

**Fig 3:**
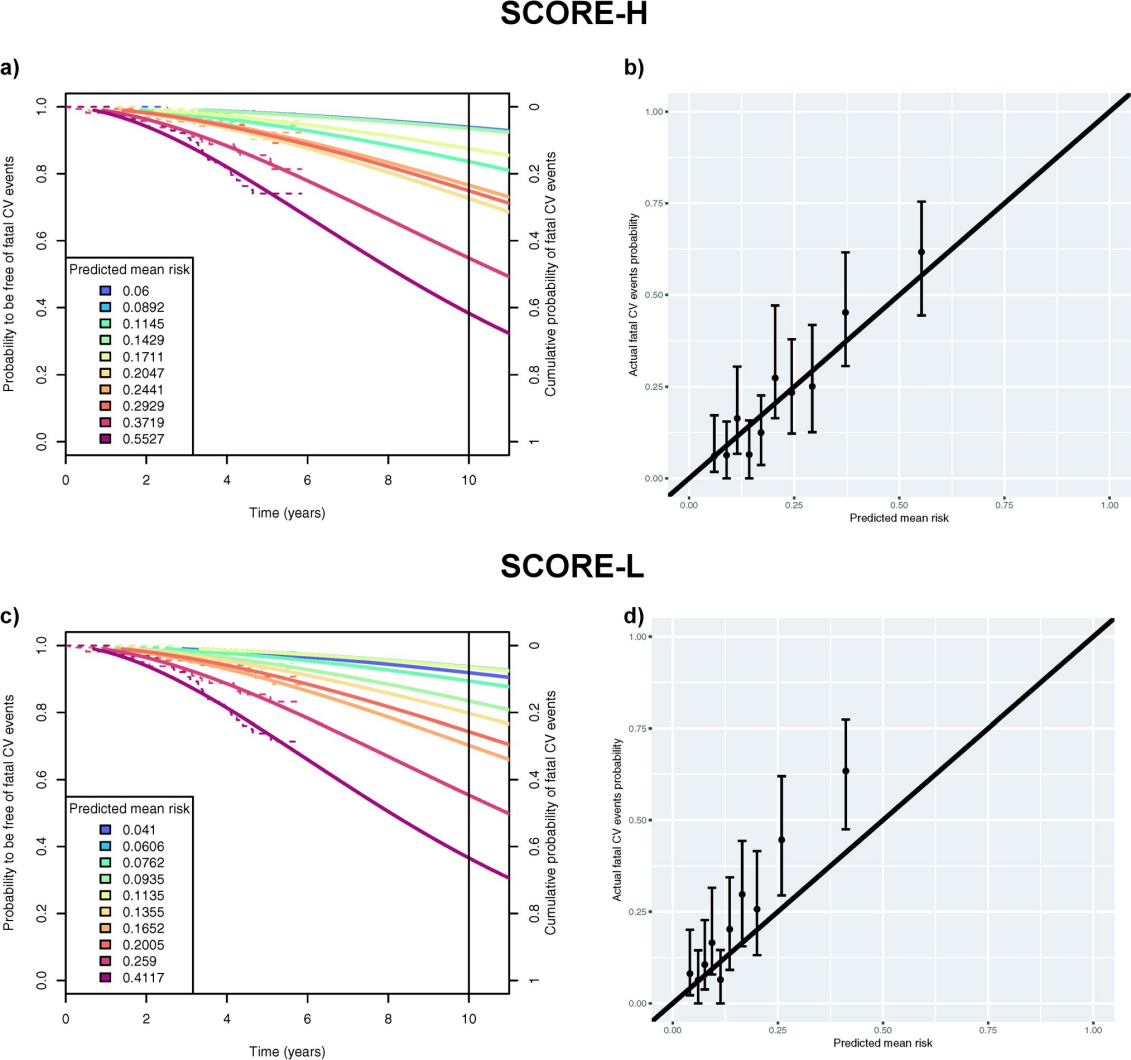
Panel a) shows both observed (Kaplan-Meier, dotted lines) and projected (Weibull regression model, solid lines) probabilities to be free of fatal cardiovascular (CV) events at a given time point grouped by deciles of risk as predicted by SCORE-H (for high-risk regions). The right y-axis scale shows the probability of the complementary event: occurrence of a fatal CV event before a given time point in the counterfactual scenario of no competing events (if we assume independent competing risks). The legend indicates the average predicted risk of having a fatal CV event within each decile group of risk. Panel b) shows the calibration plot for SCORE-H comparing the predicted mean risk (corresponding to ones in the legend of Panel a)) to the actual fatal CV event probabilities within ten years (corresponding to the intersection between the curves and the right Y-axis in Panel a)) for each decile group. We report 95% bias-corrected and accelerated bootstrapped confidence intervals. Panels c) and d) show the results as described in Panels a) and b) for SCORE-L (for low-risk regions).

For the secondary analysis, after grouping individuals according to deciles of predicted risk, we ran Weibull regression survival models. Weibull regression assumptions were fulfilled in all models (see diagnostic plots; S2 Fig in S1 File). Actual and predicted SCORE-H probabilities are displayed in Fig 3A. The corresponding calibration plot is shown in Fig 3B. Globally, SCORE-H predicted 372 expected events over ten years, and 382 actual events were projected (ratio = 0.97, chi-square = 29.7). The discrimination ability of SCORE-H as measured by the C-index was 0.72 (0.67 to 0.76).

The corresponding probabilities for SCORE-L are illustrated in Fig 3C. In eight decile groups, predicted probabilities were underestimated (Fig 3D). In total, the number of actual events projected was 384 while only 258 events were expected based on the SCORE-L (predicted/actual ratio = 0.67, chi-square = 117.6). The C-index for discrimination was found to be 0.72 (0.67 to 0.77).

**Fig 4:**
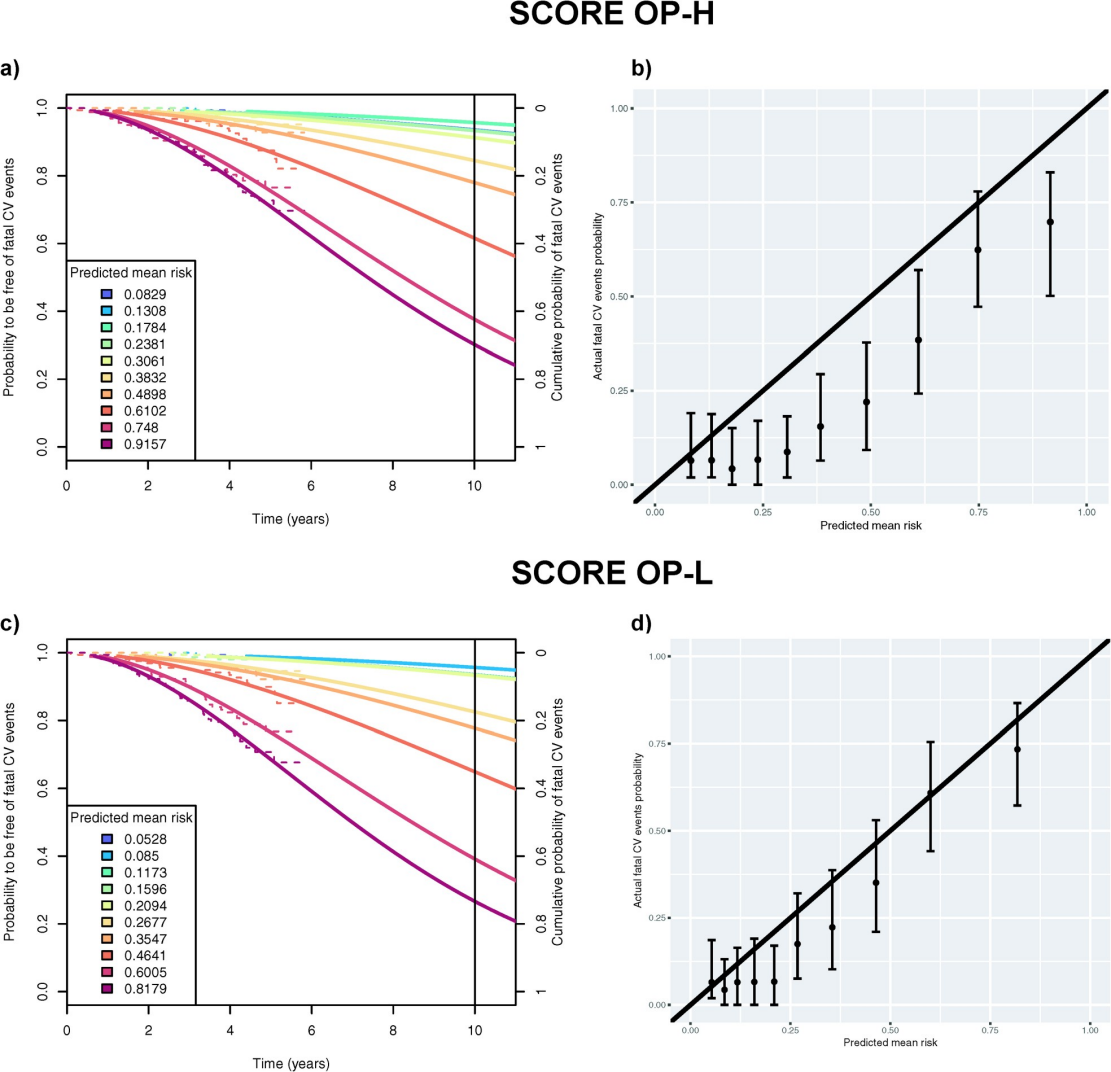
Panel a) shows both observed (Kaplan-Meier, dotted lines) and projected (Weibull regression model, solid lines) probabilities to be free of fatal cardiovascular (CV) events at a given time point grouped by deciles of risk as predicted by SCORE OP-H (for high-risk regions). The right y-axis scale shows the probability of the complementary event: occurrence of a fatal CV event before a given time point in the counterfactual scenario of no competing events (if we assume independent competing risks). The legend indicates the average predicted risk of having a fatal CV event within each decile group of risk. Panel b) shows the calibration plot for SCORE OP-H comparing the predicted mean risk (corresponding to ones in the legend of Panel a)) to the actual fatal CV event probabilities within ten years (corresponding to the intersection between the curves and the right Y-axis in Panel a)) for each decile group. We report 95% bias-corrected and accelerated bootstrapped confidence intervals. Panels c) and d) show the results as described in Panels a) and b) for SCORE OP-L (for low-risk regions).

Compared to SCORE-H, the 10-year SCORE OP-H equation designed for older persons had a higher discrimination ability in our study population (C-index = 0.79, 0.75 to 0.83). However, the SCORE OP-H overestimated risk in each decile group (Fig 4A; chi-square = 327.9). This systematic overestimation is visible in the calibration plot (Fig 4B). The SCORE OP-H predicted 677 events, while only 399 actual events were projected, a considerable overestimation (ratio = 1.70). Similarly, the 10-year version of the SCORE OP-L, despite its good discrimination ability (C-index = 0.80, 0.75 to 0.83), overestimated the risk for fatal CV events in eight decile groups (see Fig 4C and 4D). As illustrated by the calibration plot, this overestimation by SCORE OP-L was to a lesser extent than for SCORE OP-H (chi-square = 76.3). In total, SCORE OP-L predicted 519 events compared with 397 projected actual events (ratio = 1.31).

We have provided a summary of all the results previously described in Table 2.

**Table 2:** Risk scores: Measures of validity.

Risk score^a^	Predicted number of fatal cardiovascular events	Actual number of fatal cardiovascular events^b^	Predicted/ Actual ratio	Nam-D’Agostino chi-square (p-value)	C-index^c^ (95% CI)
**SCORE OP**					0.79
**high-risk**					(0.75 to 0.83)
**Regions**					
SCORE	302	142	2.13	139.16	
OP-H 5y				(p<0.001)	
SCORE	677	399	1.70	327.9	
OP-H				(p<0.001)	
**SCORE OP**					0.80
**low-risk**					(0.75 to 0.83)
**Regions**					
SCORE	215	142	1.51	39.68	
OP-L 5y				(p<0.001)	
SCORE	519	397	1.31	76.29	
OP-L				(p<0.001)	
**SCORE-H**	372	382	0.97	29.68	0.72
				(p = 0.001)	(0.67 to 0.76)
**SCORE-L**	258	384	0.67	117.63	0.72
				(p<0.001)	(0.67 to 0.77)

^a^SCORE OP[22] and SCORE[13] systems have been previously described elsewhere. H and L indicate high- and low- cardiovascular risk regions. 5y indicates 5-year risk equations. All other scores listed are 10-year versions.

^b^Weibull regression model projections beyond the observed follow-up are reported for 10-year risk scores, leading to small differences in the number of actual events. 5-year risk scores use observed Berlin Initiative Study data only using the Kaplan-Meier estimator.

^c^Risk score discrimination capability was assessed using the entire observed follow-up data.

To determine the impact of potential misclassification due to 44 fatalities with unknown cause of death information, we performed all analyses again under a ‘worst-case’ scenario assuming that all 44 individuals died due to CV reasons. In this sensitivity analysis, the SCORE-H still underestimated risk (predicted/actual ratio = 0.88), and the SCORE OP-L overestimated risk (ratio = 1.22) (S1 Table in S1 File). Consistent results were obtained upon repeating all analyses in the pseudopopulation dataset with the same size and age-sex structure of the 2010 Berlin older population, obtained by resampling (see S1 File).

We found that using SCORE OP compared to SCORE led to more individuals classified as “very-high-risk” at hypothetical thresholds beyond 10% (Table 3). For example, using a 20% cut-off, which would be reasonable given the European Society of Cardiology’s recommendation to select a threshold higher than 10% in older persons[14], results in the following percentage of participants being classified as “very-high-risk” per score: SCORE-H: 45.9% SCORE-L 25.0%, SCORE OP-H 71.2%, and SCORE OP-L 56.5%. According to the full Weibull model fitted on BIS data, 40.9% of BIS participants should be classified as “very-high-risk”.

**Table 3:** Percentage of berlin initiative study participants classified as very-high-risk^a^ based on various hypothetical thresholds of cardiovascular mortality predicted risk.

Prognostic model^b^	Predicted 10-year risk threshold				
	≥10%	≥15%	≥20%	≥25%	≥30%
SCORE-H	80.0%	62.8%	45.9%	33.2%	23.5%
SCORE-L	60.9%	39.4%	25.0%	16.3%	10.4%
SCORE OP-H	91.4%	80.4%	71.2%	62.8%	55.8%
SCORE OP-L	79.9%	67.2%	56.5%	47.6%	40.7%
Full Weibull model fitted on BIS data	58.1%	48.2%	40.9%	33.7%	28.7%

^a^In this hypothetical example, only individuals with a predicted risk higher than the threshold are considered very-high-risk persons. This simplification is only intended to illustrate possible clinical implications of the use of prognostic tools.

^b^SCORE[13] and SCORE OP[22] risk scores have been previously described elsewhere. H and L indicate high- and low- cardiovascular risk regions. The full Weibull model fitted on BIS data includes sex, systolic blood pressure, total cholesterol, HDL cholesterol, smoking status, diabetes and age as covariates and cardiovascular fatal events as the outcome to give a near description of the projected reality at 10 years for our cohort.

**Fig 5:**
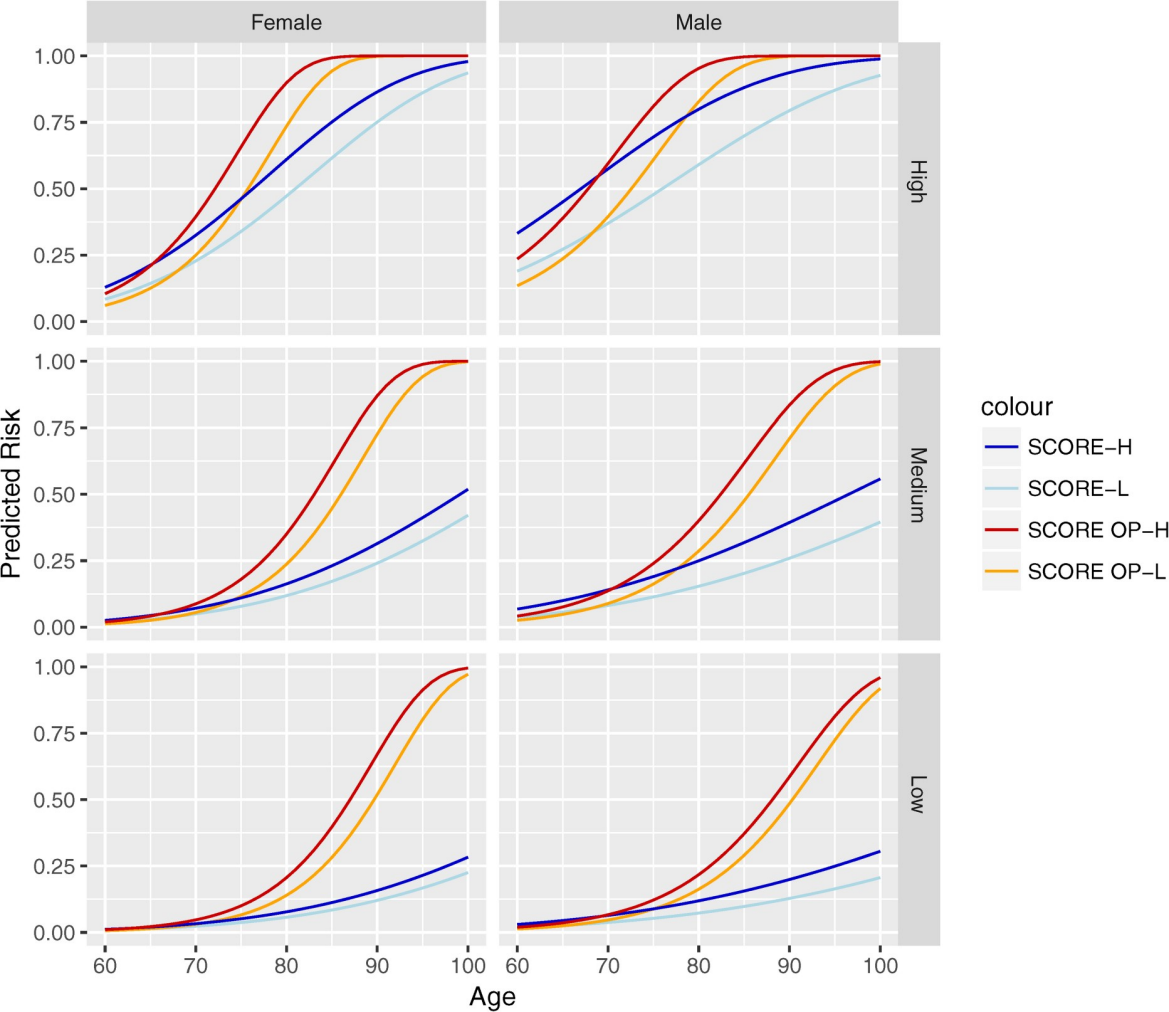
We compared SCORE-L, SCORE-H, SCORE OP-H and SCORE OP-L predicted 10-year risks for six risk profiles; high-, medium- and low-risk for females and males. The high-risk profile was constructed using data from a hypothetical diabetic, current smoker with a systolic blood pressure of 180 mmHg, total cholesterol level of 8 mmol/l, and HDL cholesterol level of 1 mmol/l. The medium-risk profile was created using mean values for all variables based on the baseline characteristics of Berlin Initiative Study participants (see Table 1). The low-risk profile was constructed using data from a hypothetical non-diabetic, current non-smoker with a systolic blood pressure of 120 mmHg, total cholesterol level of 4 mmol/l, and HDL cholesterol level of 2 mmol/l. The high- and low-risk reference values for systolic blood pressure and total cholesterol were taken from the minimum and maximum values reported in the SCORE chart.

To further explore differences in performance of the two score systems among older persons, we compared fatal CV event risk predicted by the two risk scores for six hypothetical risk profiles based on risk factors. We created high-, medium-, and low-risk examples for females and males across the entire age spectrum from 60 to 100 years (see Fig 5). We found that SCORE OP predicted higher risks compared to SCORE in female individuals aged ≥75 and in male individuals aged ≥78.

## Discussion

In this prospective, population-based study of Berlin older individuals, the SCORE OP 5y substantially overestimated the true risk of fatal CV events. In the 10-year comparison, interestingly, the SCORE equation recommended for persons aged from 40 to 65, showed better calibration than the SCORE OP, developed specifically for persons aged from 65 to 80[22]. The SCORE OP did, however, demonstrate slightly superior discrimination capabilities as assessed by the C-index, likely attributable to the higher number of included risk factors.

Because the prevalence of CV risk factors and their estimated effects on CV outcomes are known to change with increasing age[20,32], the SCORE OP was developed to correct for a suspected overestimation of fatal CV event risk as predicted by the original SCORE in persons aged ≥65[22]. The SCORE development dataset was comprised largely of middle-aged individuals (only three cohorts out of 12 included participants aged ≥70, and no cohort included individuals aged >80). Because of this, it was hypothesized that the estimated prediction model beta coefficients were inappropriately large in magnitude, likely overemphasizing the contribution of these risk factors to the predicted risk in older persons[20,22,32,33]. Since the levels of traditional CV risk factors are known to be elevated in older persons, the use of inappropriately large coefficients for this population was expected to result in substantial overestimation of the true fatal CV event risk using the SCORE[22].

The SCORE OP is based on re-estimated beta coefficients using data from people aged ≥65 also including additional parameters (diabetes and HDL) in an attempt to correct the expected overestimation. Based on this rationale, the use of SCORE OP is currently suggested as an alternative by the European Society of Cardiology and European Society of Hypertension guidelines for the management of arterial hypertension in older individuals (aged ≥65)[34].

The idea that the SCORE gives higher estimates of risk compared to the SCORE OP among older persons was reinforced by findings from a cross-sectional study in Spain[35,36] and the charts comparison presented in the SCORE OP development paper (p.8)[22]. However, these two comparisons were based solely on the 65–69 age group. No attempt was made to compare predictions across *all* ages 65 and above.

In our cohort comprised of older persons, we found that SCORE OP yielded higher risk estimates than SCORE (with mathematical correction). The same result was found in the only published external validation of the SCORE OP[24]. While the authors attributed this inconsistency with the previous cross-sectional study[35,36] to a difference in the methods used, we think it may have arisen because of a difference in age composition of the participants. Upon comparing the scores designed for low-risk regions, we confirm that in the age group 65–68, the SCORE-L yields higher risk estimates than the SCORE OP-L for medium- and high-risk individuals (see Fig 5). However, this is not true for any of the risk profiles in individuals aged ≥70; in these individuals, the risk estimated by SCORE OP-L consistently exceeds that of SCORE-L. We observed a similar behavior upon comparing the high-risk region risk scores (Fig 5).

Moreover, we found a lower transportability of the SCORE OP compared to the original SCORE. This is likely explained by a difference in the distribution of unmeasured risk factors and baseline CV fatal risk in our cohort compared to the SCORE OP development dataset. The SCORE OP development dataset was composed of 85% individuals from Norway and included no German cohorts, while the SCORE development dataset included a German cohort and a balanced distribution of individuals from several European countries. Differences in the definition of CV risk factors included in the models or in the true underlying hazard ratios may have led to the unsuitability of the SCORE OP coefficients. For example, in the SCORE OP development dataset, included cohort prevalences of diabetes mellitus ranged from 6% to 7% in females and 4% to 7% in males[22]. These prevalences are considerably lower than the diabetes mellitus prevalences observed in the Berlin Initiative Study (23% in females and 27% in males, which align with German prevalence figures among older persons[37]) and it is likely a difference in the definition of diabetes contributed to the observed low transportability of the SCORE OP.

We found the best performing risk score among older persons in Berlin was the SCORE-H developed for high-risk regions despite that Germany’s fatal CV risk is considered to be between that of high- and low-risk regions, with a tendency towards the latter[27]. The fact that the SCORE-L seems more appropriate in middle-aged persons while the SCORE-H seems better suited among older persons suggests that in the Berlin population, the observed difference in risk prediction is likely explained by differences in baseline survivorship functions (determined by age, sex, unmeasured risk factors) of these age groups rather than by differences in the coefficients of classical risk factors such as cholesterol, blood pressure and smoking. In fact, SCORE developers used the same coefficients in both regional versions.

In general, the performance of the SCORE equations was surprisingly good, especially considering that this risk score was developed in 2003 using data from cohort studies with recruitment periods between 1967 and 1991 and that the incidence and treatment strategies for CV diseases changed substantially over the last decades[38].

Recently, the SCORE OP was subjected to external validation for the first time in a cohort of 6,590 older individuals aged between 65 and 79 living in Norfolk, UK[24]. Their results about the transportability of the SCORE OP and SCORE among older persons were divergent compared to ours. In this UK population, the SCORE OP showed “excellent calibration”, performing better than the SCORE, despite showing low discrimination ability[24]. Interestingly, in this UK cohort, diabetes prevalence was extremely low, around 3%[24], likely because this information was only self-reported. This observation provides additional support for our aforementioned argument that the definition of diabetes plays a crucial role in the transportability of the SCORE OP. Regional differences and the age of the cohort, overall younger than our study participants, may also have contributed to the discrepancies.

Finally, the SCORE and SCORE-OP were developed without accounting for possible competing events, thus, our calibration used a consistent approach; neither the Weibull model projections nor the Kaplan-Meier estimates accounted for competing events. We acknowledge, however, that competing risks do pose a large problem for practical use among older persons, in whom competing fatal events are common. For this reason, methods have been suggested for the development of local, updated, recalibrated scores that can be used to inform regional risk prediction accounting for mortality due to other causes[28].

### Study strengths and limitations

Strengths include the prospective design, population-based setting, and availability of comprehensive health-related information, providing unique insights into the health of the very old, a population often excluded from larger studies. Death information is considered to be complete and was obtained from the Berlin death certificates archive and supplemented with information from medical records. Specific cause of death information was consistently extracted when available. Furthermore, a total of 118 fatal CV endpoints were recorded during observed follow-up, exceeding the minimum amount needed to properly validate a 10-year prognostic model over the entire time span (at least 100)[39].

Some limitations should be considered when interpreting our findings. First, we compared predicted probabilities to projected ones since the Berlin Initiative Study follow-up data were available for less than 10 years. However, the Weibull diagnostic plots indicate fulfillment of the assumptions of all projection models, and our analyses predicting 5-year risk using observed data were consistent with the findings using 10-year projected probabilities and thus confirm our results. A minor drawback to our approach is that the number of “actual” events is not constant across calibration assessments because it depends on decile groupings of participants, which differed for each risk score. However, these “actual” event numbers did not differ substantially (range: 382 to 399).

Second, the reliability of cause of death information on death certificates is known to be error-prone, especially among older persons. However, we believe this potential misclassification is similar for most population-based settings and was also present in the risk score development studies[22].The impact of unknown or unavailable cause of death information as demonstrated by our “worst-case” sensitivity analysis was negligible.

Furthermore, all of the original SCORE endpoints are well-represented in our definition, with the exception of non-aortic aneurysms, which are very rare events.

Finally, the exclusion of subjects with previous history of myocardial infarction was based on self-reported information. However, the self-reported nature of this information in our study is unlikely to be problematic, since this exclusion criterion was introduced during the SCORE development only to ensure overall CV health in the considered sample and not for the purpose of excluding participants not at risk for the outcome.

Nonetheless, we suggest against excluding people with a predefined CV event from future prognostic models developed to predict CV mortality risk, as this limits prediction score usability. This is a particularly important consideration among older persons, in whom prevalent non-fatal CV events are common.

### Implications and suggestions for future research

This external validation study shows that the SCORE OP overestimates CV mortality risk among Berlin older persons, which ultimately leads to the classification of more individuals to higher levels of CV mortality risk at most hypothetical very-high-risk thresholds, above which intervention strategies would be indicated. We believe our results may have important implications since overestimation of risk in these individuals may lead to overtreatment in a potentially vulnerable population already prone to polypharmacy[40], which is known to lead to adverse drug events or interactions and increase health care costs[41]. Our results underscore the importance of external validation of prediction tools before clinical use. Mass medicalization may result from an overestimation due to lack of transportability of these tools or from setting too low of a risk threshold[42]. This potential danger is illustrated by the higher number of BIS participants who would be classified as very-high-risk for 10-year fatal CV risk according to the SCORE OP compared to the reality.

In our external validation study, the original SCORE developed for high-risk regions performed best in older persons living in Berlin. Our findings are very different from the ones obtained in the first SCORE OP external validation study conducted among older adults in the UK; however, both studies do not support the use of SCORE OP in clinical practice. Therefore, the challenge of finding a valid tool for profiling risk among older European individuals may not yet be solved.

## Supporting information

S1 File (PDF)

## References

[1] Nichols M, Townsend N, Scarborough P, Rayner M. European cardiovascular disease statistics. European Heart Network; 2012 [cited 29 Oct 2018]. Available: http://www.ehnheart.org/component/attachments/attachments.html?task=download&folder=publications&id=1435

[2] WHO. cardiovascular diseases (CVDs). 2016 [cited 29 Oct 2018]. Available: http://www.who.int/news-room/fact-sheets/detail/cardiovascular-diseases-(cvds)

[3] Vartiainen E, Puska P, Pekkanen J, Tuomilehto J, Jousilahti P. Changes in risk factors explain changes in mortality from ischaemic heart disease in Finland. BMJ. 1994;309: 23–27. 10.1136/bmj.309.6946.23 8044063 PMC2542620

[4] WHO Europe. cardiovascular disease data and statistics. 1 Aug 2018 [cited 29 Oct 2018]. Available: http://www.euro.who.int/en/health-topics/noncommunicable-diseases/cardiovascular-diseases/data-and-stat istics

[5] Vartiainen E, Sarti C, Tuomilehto J, Kuulasmaa K. Do changes in cardiovascular risk factors explain changes in mortality from stroke in Finland? BMJ. 1995;310: 901–904. 10.1136/bmj.310.6984.901 7719179 PMC2549289

[6] Menotti A, Puddu PE, Kromhout D, Kafatos A, Tolonen H. Coronary heart disease mortality trends during 50 years as explained by risk factor changes: The European cohorts of the Seven Countries Study. Eur J Prev Cardiol. 2019; 2047487318821250. 10.1177/2047487318821250 30614262

[7] Stewart J, Manmathan G, Wilkinson P. Primary prevention of cardiovascular disease: A review of contemporary guidance and literature. JRSM Cardiovasc Dis. 2017;6: 2048004016687211 10.1177/2048004016687211 28286646 PMC5331469

[8] Collins DRJ, Tompson AC, Onakpoya IJ, Roberts N, Ward AM, Heneghan CJ. Global cardiovascular risk assessment in the primary prevention of cardiovascular disease in adults: systematic review of systematic reviews. BMJ Open. 2017;7: e013650 10.1136/bmjopen-2016-013650 28341688 PMC5372072

[9] Damen JAAG, Hooft L, Schuit E, Debray TPA, Collins GS, Tzoulaki I, et al Prediction models for cardiovascular disease risk in the general population: systematic review. BMJ. 2016;353: i2416 10.1136/bmj.i2416 27184143 PMC4868251

[10] Kannel WB, McGee D, Gordon T. A general cardiovascular risk profile: the Framingham Study. Am J Cardiol. 1976;38: 46–51. 10.1016/0002-9149(76)90061-8 132862

[11] D’Agostino RB Sr, Vasan RS, Pencina MJ, Wolf PA, Cobain M, Massaro JM, et al General cardiovascular risk profile for use in primary care: the Framingham Heart Study. Circulation. 2008;117: 743–753. 10.1161/CIRCULATIONAHA.107.699579 18212285

[12] Wilson PW, D’Agostino RB, Levy D, Belanger AM, Silbershatz H, Kannel WB. Prediction of coronary heart disease using risk factor categories. Circulation. 1998;97: 1837–1847. 10.1161/01.cir.97.18.1837 9603539

[13] Conroy RM, Pyörälä K, Fitzgerald AP, Sans S, Menotti A, De Backer G, et al Estimation of ten-year risk of fatal cardiovascular disease in Europe: the SCORE project. Eur Heart J. 2003;24: 987–1003. 10.1016/s0195-668x(03)00114-3 12788299

[14] Authors/Task Force Members:, Piepoli MF, Hoes AW, Agewall S, Albus C, Brotons C, et al 2016 European Guidelines on cardiovascular disease prevention in clinical practice: The Sixth Joint Task Force of the European Society of Cardiology and Other Societies on Cardiovascular Disease Prevention in Clinical Practice (constituted by representatives of 10 societies and by invited experts) Developed with the special contribution of the European Association for Cardiovascular Prevention & Rehabilitation (EACPR). Atherosclerosis. 2016;252: 207–274. 10.1016/j.atherosclerosis.2016.05.037 27664503

[15] Panagiotakos DB, Fitzgerald AP, Pitsavos C, Pipilis A, Graham I, Stefanadis C. Statistical modelling of 10-year fatal cardiovascular disease risk in Greece: the HellenicSCORE (a calibration of the ESC SCORE project). Hellenic J Cardiol. 2007;48: 55–63. 17489342

[16] Marques-Vidal P, Rodondi N, Bochud M, Pécoud A, Hayoz D, Paccaud F, et al Predictive accuracy and usefulness of calibration of the ESC SCORE in Switzerland. Eur J Cardiovasc Prev Rehabil. 2008;15: 402–408. 10.1097/HJR.0b013e3282fb040f 18677163

[17] van Dis I, Kromhout D, Geleijnse JM, Boer JMA, Verschuren WMM. Evaluation of cardiovascular risk predicted by different SCORE equations: the Netherlands as an example. Eur J Cardiovasc Prev Rehabil. 2010;17: 244–249. 10.1097/HJR.0b013e328337cca2 20195155

[18] De Bacquer D, De Backer G. Predictive ability of the SCORE Belgium risk chart for cardiovascular mortality. Int J Cardiol. 2010;143: 385–390. 10.1016/j.ijcard.2009.03.101 19386372

[19] Rodondi N, Locatelli I, Aujesky D, Butler J, Vittinghoff E, Simonsick E, et al Framingham risk score and alternatives for prediction of coronary heart disease in older adults. PLoS One. 2012;7: e34287 10.1371/journal.pone.0034287 22470551 PMC3314613

[20] Cooney MT, Dudina AL, Graham IM. Value and limitations of existing scores for the assessment of cardiovascular risk: a review for clinicians. J Am Coll Cardiol. 2009;54: 1209–1227. 10.1016/j.jacc.2009.07.020 19778661

[21] Koller MT, Steyerberg EW, Wolbers M, Stijnen T, Bucher HC, Hunink MGM, et al Validity of the Framingham point scores in the elderly: results from the Rotterdam study. Am Heart J. 2007;154: 87–93. 10.1016/j.ahj.2007.03.022 17584559

[22] Cooney MT, Selmer R, Lindman A, Tverdal A, Menotti A, Thomsen T, et al Cardiovascular risk estimation in older persons: SCORE OP. Eur J Prev Cardiol. 2016;23: 1093–1103. 10.1177/2047487315588390 26040999

[23] Mortensen MB, Afzal S, Nordestgaard BG, Falk E. The high-density lipoprotein-adjusted SCORE model worsens SCORE-based risk classification in a contemporary population of 30 824 Europeans: the Copenhagen General Population Study. Eur Heart J. 2015;36: 2446–2453. 10.1093/eurheartj/ehv251 26082084 PMC4576144

[24] Verweij L, Peters RJG, Scholte Op Reimer WJM, Boekholdt SM, Luben RM, Wareham NJ, et al Validation of the Systematic COronary Risk Evaluation—Older Persons (SCORE-OP) in the EPIC-Norfolk prospective population study. Int J Cardiol. 2019;293: 226–230. 10.1016/j.ijcard.2019.07.020 31324398

[25] Schaeffner ES, van der Giet M, Gaedeke J, Tölle M, Ebert N, Kuhlmann MK, et al The Berlin initiative study: the methodology of exploring kidney function in the elderly by combining a longitudinal and cross-sectional approach. Eur J Epidemiol. 2010;25: 203–210. 10.1007/s10654-010-9424-x 20094758

[26] Ebert N, Jakob O, Gaedeke J, van der Giet M, Kuhlmann MK, Martus P, et al Prevalence of reduced kidney function and albuminuria in older adults: the Berlin Initiative Study. Nephrol Dial Transplant. 2017;32: 997–1005. 10.1093/ndt/gfw079 27190381

[27] Rücker V, Keil U, Fitzgerald AP, Malzahn U, Prugger C, Ertl G, et al Predicting 10-Year Risk of Fatal Cardiovascular Disease in Germany: An Update Based on the SCORE-Deutschland Risk Charts. PLoS One. 2016;11: e0162188 10.1371/journal.pone.0162188 27612145 PMC5017762

[28] Støvring H, Harmsen CG, Wisløff T, Jarbøl DE, Nexøe J, Nielsen JB, et al A competing risk approach for the European Heart SCORE model based on cause-specific and all-cause mortality. Eur J Prev Cardiol. 2013;20: 827–836. 10.1177/2047487312445425 22498473

[29] D’Agostino RB, Nam B-H. Evaluation of the Performance of Survival Analysis Models: Discrimination and Calibration Measures Handbook of Statistics. Elsevier; 2003 pp. 1–25.

[30] Therneau T, Atkinson E. Concordance. The Comprehensive R Archive Network; 2019 3 Available: https://cran.r-project.org/web/packages/survival/vignettes/concordance.pdf

[31] Berlin Brandenburg Amt Für Statistik. Statistisches Informationssystem Berlin Brandenburg (StatIS-BBB). [cited 19 Mar 2019]. Available: www.statistik-berlin-brandenburg.de

[32] Cooney MT, Dudina A, D’agostino R, Graham IM. Cardiovascular risk-estimation systems in primary prevention: do they differ? Do they make a difference? Can we see the future? Circulation. 2010 Available: https://www.ahajournals.org/doi/abs/10.1161/circulationaha.109.852756 10.1161/CIRCULATIONAHA.109.852756 20644026

[33] Catapano AL, Graham I, De Backer G, Wiklund O, Chapman MJ, Drexel H, et al 2016 ESC/EAS Guidelines for the Management of Dyslipidaemias: The Task Force for the Management of Dyslipidaemias of the European Society of Cardiology (ESC) and European Atherosclerosis Society (EAS) Developed with the special contribution of the European Association for Cardiovascular Prevention & Rehabilitation (EACPR). Atherosclerosis. 2016;253: 281–344. 10.1016/j.atherosclerosis.2016.08.018 27594540

[34] Williams B, Mancia G, Spiering W, Agabiti Rosei E, Azizi M, Burnier M, et al 2018 ESC/ESH Guidelines for the management of arterial hypertension. Eur Heart J. 2018;39: 3021–3104. 10.1093/eurheartj/ehy339 30165516

[35] Brotons C, Moral I, Fernández D, Cuixart L, Soteras A, Puig M. Assessment of the New SCORE OP Cardiovascular Risk Charts in Patients Older Than 65 Years. Rev Esp Cardiol. 2016;69: 981–983. 10.1016/j.rec.2016.04.049 27474480

[36] Brotons C, Moral I, Fernández D, Cuixart L, Muñoz A, Soteras A, et al [Clinical consequences of using the new cardiovascular risk tables SCORE OP in patients aged over 65 years]. Med Clin. 2016;147: 381–386. 10.1016/j.medcli.2016.06.035 27575527

[37] Tamayo T, Brinks R, Hoyer A, Kuß OS, Rathmann W. The Prevalence and Incidence of Diabetes in Germany. Dtsch Arztebl Int. 2016;113: 177–182. 10.3238/arztebl.2016.0177 27118665 PMC4850517

[38] Mortensen MB, Falk E. Limitations of the SCORE-guided European guidelines on cardiovascular disease prevention. Eur Heart J. 2017;38: 2259–2263. 10.1093/eurheartj/ehw568 27941016 PMC5946870

[39] Collins GS, Ogundimu EO, Altman DG. Sample size considerations for the external validation of a multivariable prognostic model: a resampling study. Stat Med. 2016;35: 214–226. 10.1002/sim.6787 26553135 PMC4738418

[40] O Riordan D, Aubert CE, Walsh KA, Van Dorland A, Rodondi N, Du Puy RS, et al Prevalence of potentially inappropriate prescribing in a subpopulation of older European clinical trial participants: a cross-sectional study. BMJ Open. 2018;8: e019003 10.1136/bmjopen-2017-019003 29567842 PMC5875647

[41] Maher RL, Hanlon J, Hajjar ER. Clinical consequences of polypharmacy in elderly. Expert Opin Drug Saf. 2014;13: 57–65. 10.1517/14740338.2013.827660 24073682 PMC3864987

[42] Lancet T. Statins for millions more? Elsevier; 2014 10.1016/s0140-6736(14)60240-3 24560042

