# Correction: Performance of risk prediction scores for cardiovascular mortality in older persons: External validation of the SCORE OP and appraisal

**DOI:** 10.1371/journal.pone.0233051

**Published:** 2020-05-06

**Authors:** Marco Piccininni, Jessica L. Rohmann, Dörte Huscher, Nina Mielke, Natalie Ebert, Giancarlo Logroscino, Elke Schäffner, Tobias Kurth

There are errors in the layout of Table 2. Please see the correct Table 2 here.

**Table 2:** Risk scores: measures of validity.

Risk score^a^	Predicted number of fatal cardiovascular events	Actual number of fatal cardiovascular events^b^	Predicted/ Actual ratio	Nam-D’Agostino chi-square (p-value)	C-index^c^ (95% CI)
**SCORE OP** **high-risk regions**					0.79 (0.75 to 0.83)
SCORE OP-H 5y	302	142	2.13	139.16 (p<0.001)	
SCORE OP-H	677	399	1.70	327.9 (p<0.001)	
**SCORE OP** **low-risk regions**					0.80 (0.75 to 0.83)
SCORE OP-L 5y	215	142	1.51	39.68 (p<0.001)	
SCORE OP-L	519	397	1.31	76.29 (p<0.001)	
**SCORE-H**	372	382	0.97	29.68 (p = 0.001)	0.72 (0.67 to 0.76)
**SCORE-L**	258	384	0.67	117.63 (p<0.001)	0.72 (0.67 to 0.77)

^a^SCORE OP[22] and SCORE[13] systems have been previously described elsewhere. H and L indicate high- and low- cardiovascular risk regions. 5y indicates 5-year risk equations. All other scores listed are 10-year versions.

^b^Weibull regression model projections beyond the observed follow-up are reported for 10-year risk scores, leading to small differences in the number of actual events. 5-year risk scores use observed Berlin Initiative Study data only using the Kaplan-Meier estimator.

^c^Risk score discrimination capability was assessed using the entire observed follow-up data.
