# Supplementary material for: Performance of risk prediction scores for cardiovascular mortality in older persons: External validation of the SCORE OP and appraisal
Source: PLoS One. 2020 Apr 9;15(4):e0231097. doi: 10.1371/journal.pone.0231097 (PMC7144969; doi:10.1371/journal.pone.0231097)
Supplement: S1 File — (PDF) [file pone.0231097.s001.pdf]

Supporting Information for “Performance of risk prediction scores for cardiovascular mortality in older persons: External validation of the SCORE OP and appraisal”

Supporting Information:

- S1 Methods and Results.
- S1 Table. Sensitivity analysis: ‘worst-case’ scenario for classification of unknown cause of death.
- S1 Fig. Correlation plot of all included risk scores.
- S2 Fig. Diagnostic plots for Weibull regression models used in projections.
- S3 Fig. Sensitivity Analyses- additional calibration plots.
- S1 R Code.

S1 Methods and Results.

Sensitivity Analysis: pseudopopulation representative of Berlin 2010 general population

Calibration of the six risk score equations (SCORE-H, SCORE-L, SCORE OP-H, SCORE OP-L, SCORE OP-H 5y, SCORE OP-L 5y) were additionally assessed in a bootstrapped dataset, in which BIS individuals were resampled from each age and sex strata in order to create a pseudopopulation exactly representative in terms of size as well as age and sex structure of the Berlin population aged 70 or older at the time of BIS enrollment using 2010 data from the Berlin region.[1]

The aim of this sensitivity analysis was to provide an indication of the robustness of our results and to provide a rough idea of how the risk scores behave in a large-scale population, such as that of Berlin.

In order to account for the variability introduced by the resampling procedure, we created five unique bootstrapped datasets and re-ran the analyses in each of them. We also re-ran the sensitivity analyses in these bootstrapped datasets under the extreme ‘worst-case’ assumption, in which all unknown causes of death were classified as fatal CV events. Overall, these results were consistent with the main findings using the BIS study population; overestimation of fatal CV event risk by the SCORE OP was observed and the SCORE showed comparatively better performance. The calibration plots (12 in total) of one resampled dataset analysis are shown in Supplemental Fig. 3.

Supplemental Tables and Figures

S1 Table. Sensitivity analysis: 'worst-case' scenario for classification of unknown cause of death

Risk score[a]	Predicted number of fatal cardiovascular events	Actual number of fatal cardiovascular events[b]	Predicted to Actual ratio	Nam-D'Agostino chi-square (p-value)	C-index[c] (95% CI)
**SCORE OP high risk regions**					0.79 (0.75 to 0.82)
SCORE OP-H 5y	302	187	1.62	73.24 (p<0.001)	
SCORE OP-H	677	428	1.58	249.85 (p<0.001)	
**SCORE OP low risk regions**					0.79 (0.75 to 0.82)
SCORE OP-L 5y	215	187	1.15	11.52 (p=0.318)	
SCORE OP-L	519	426	1.22	47.70 (p<0.001)	
**SCORE-H**	372	420	0.88	23.69 (p=0.008)	0.71 (0.67 to 0.75)
**SCORE-L**	258	422	0.61	157.79 (p<0.001)	0.72 (0.67 to 0.75)

Under this extreme 'worst case' assumption, all deaths of unknown cause were considered as being of cardiovascular nature.

[a] SCORE OP[3] and SCORE[2] scores have been previously described elsewhere. 5y indicates 5-year risk equations. H and L indicate high- and low- cardiovascular risk regions. Unless otherwise specified, 10-year risk equations were used.

[b]Weibull regression model projections beyond the observed follow-up are reported for 10-year risk scores, leading to small differences in the number of actual events. 5-year risk scores use observed Berlin Initiative Study data only using the Kaplan-Meier estimator.

[c]Risk score discrimination capability was assessed using observed follow-up data only.

S1 Fig. Correlation plot of all included risk scores.

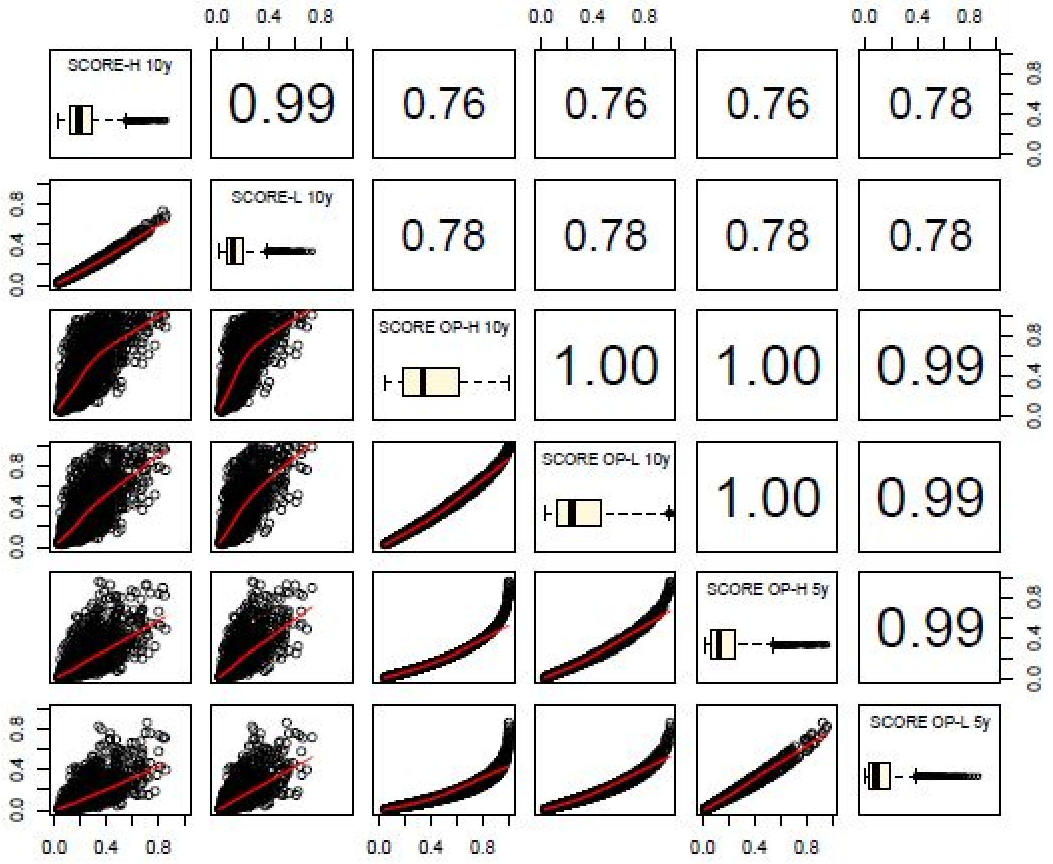

On the diagonal, boxplots of predicted risk for each score are shown. Below the diagonal, we show pairwise scatterplots of risk as predicted by the risk equations. Above the diagonal, pairwise Spearman correlation coefficients are displayed (font size proportional to correlation magnitude).

S2 Fig. Diagnostic plots for Weibull regression models used in projections.

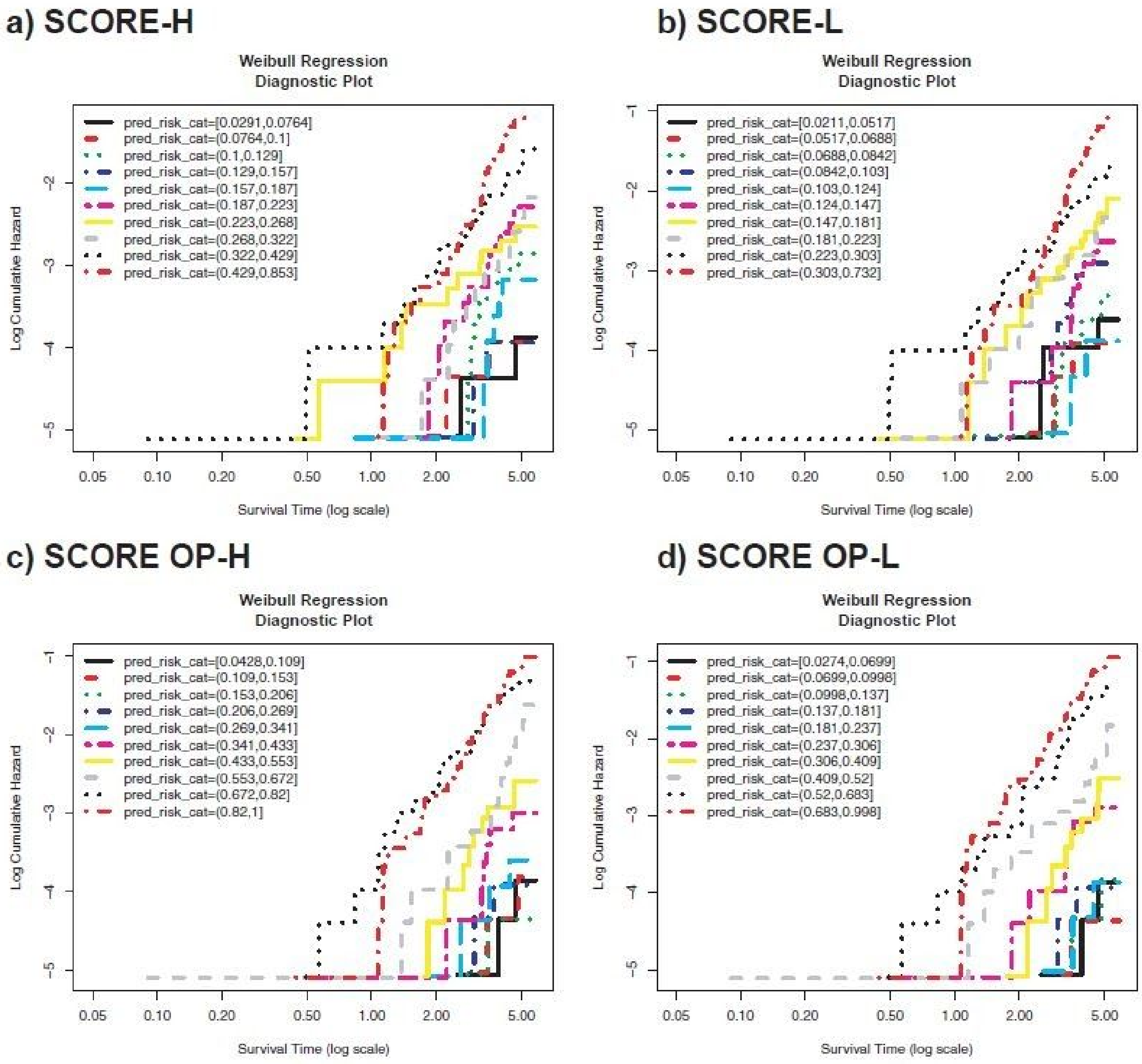

We created diagnostic plots to check the distributional assumptions of the Weibull regression models used to project a) SCORE-H, b) SCORE-L, c) SCORE OP-H, and d) SCORE OP-L fatal CV event probabilities beyond the follow-up period. Plots show the logarithm of the cumulative hazard against the logarithm of the survival time for each decile group. Roughly straight and parallel lines indicate Weibull and proportional hazards assumptions hold.

S3 Fig. Sensitivity Analyses- additional calibration plots.

a) SCORE OP-H 5y

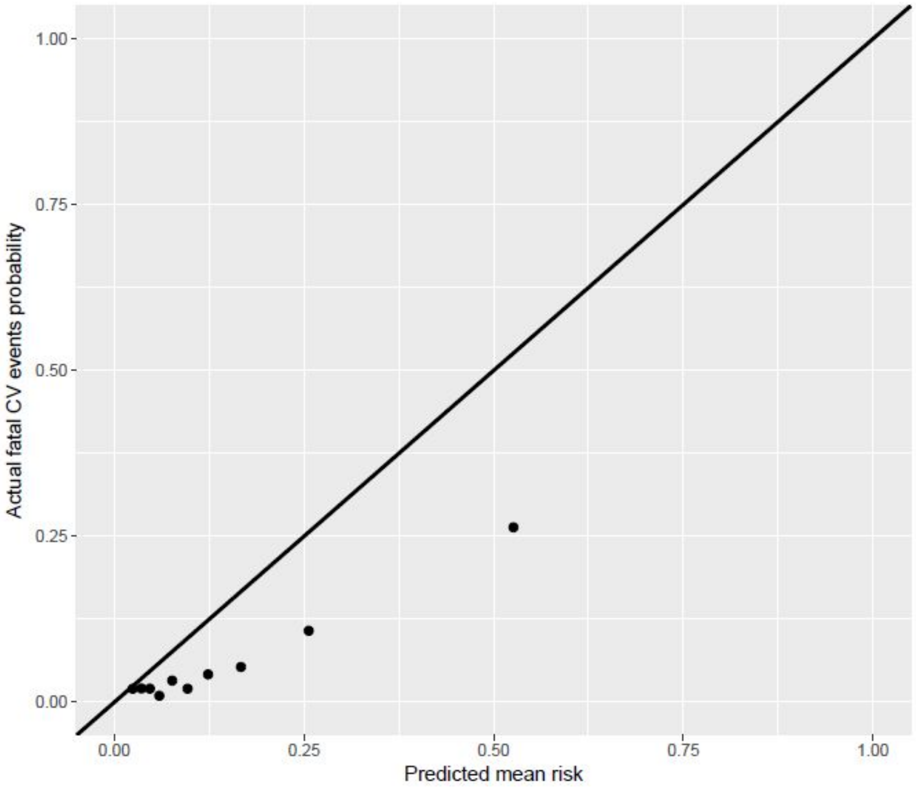

b) SCORE OP-L 5y

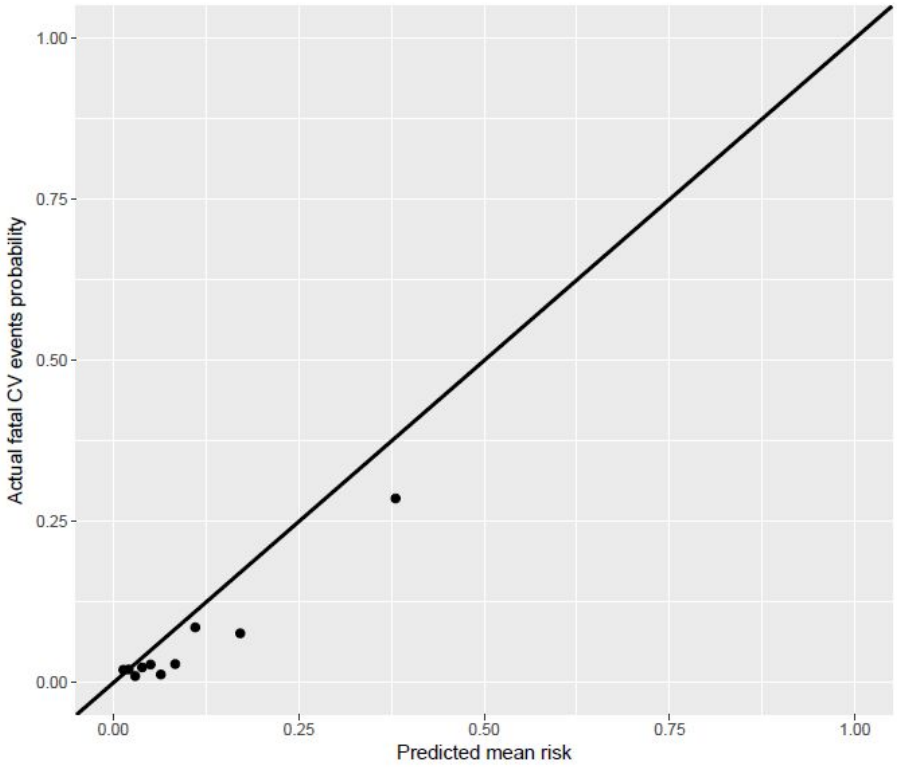

c) SCORE-H

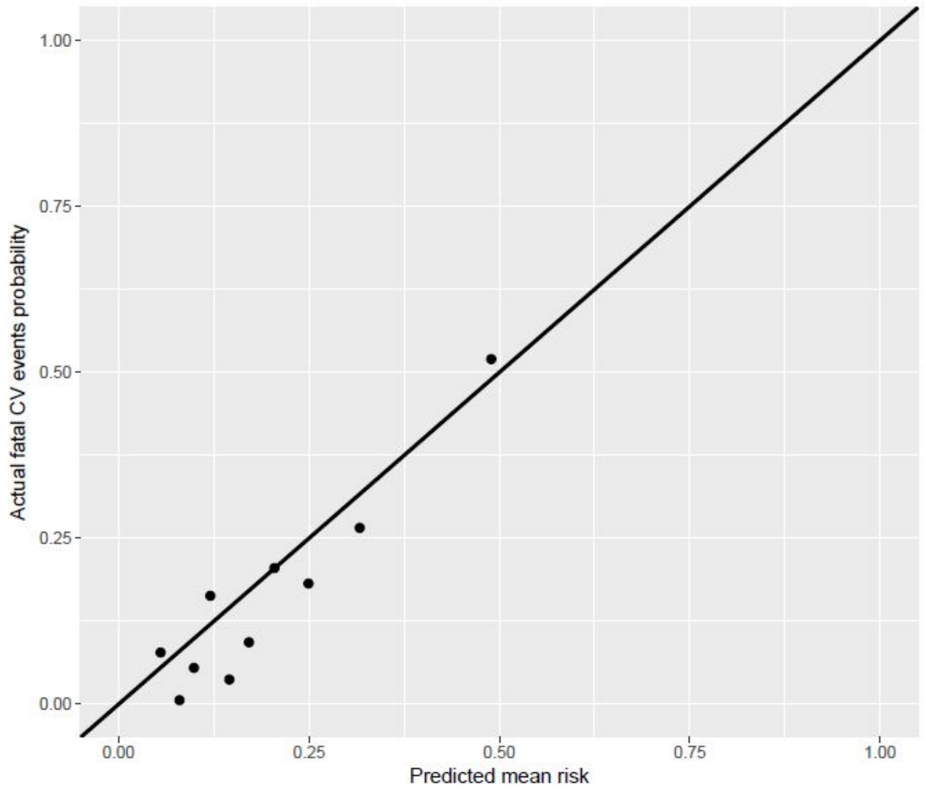

d) SCORE-L

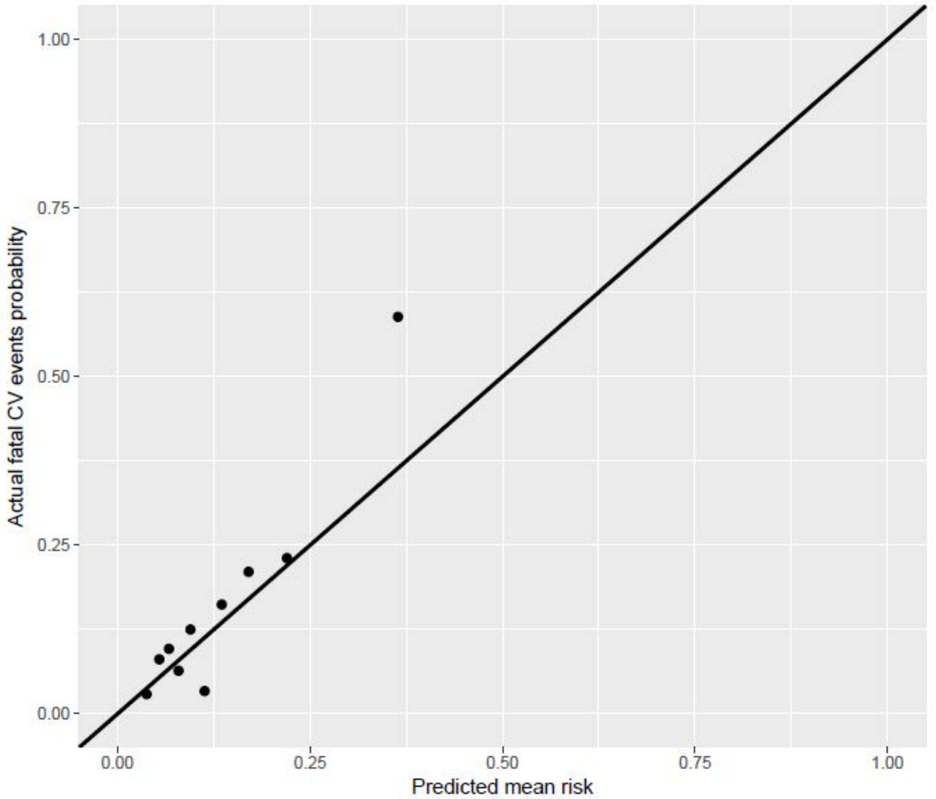

e) SCORE OP-H

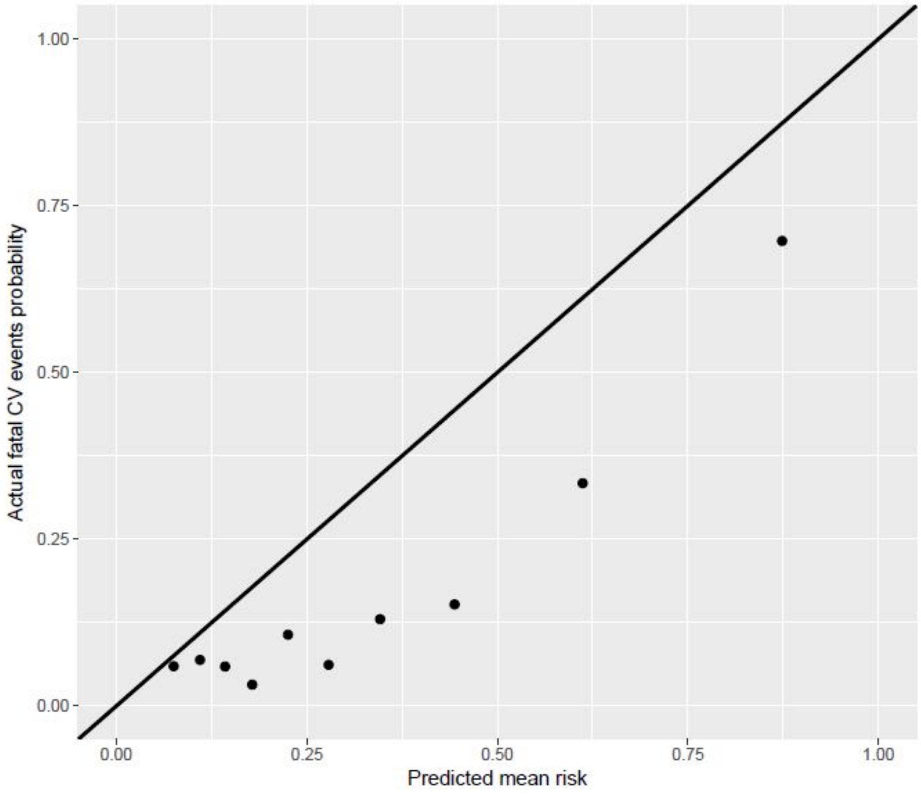

f) SCORE OP-L

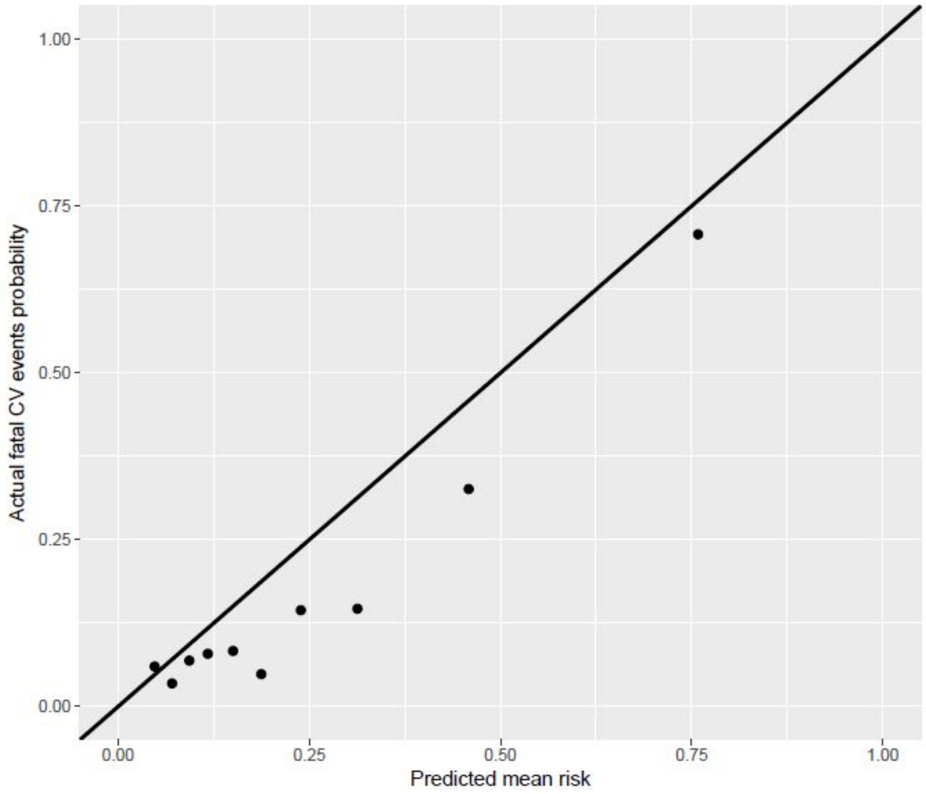

g) SCORE OP-H 5y 'worst-case' scenario for classification of unknown cause of death

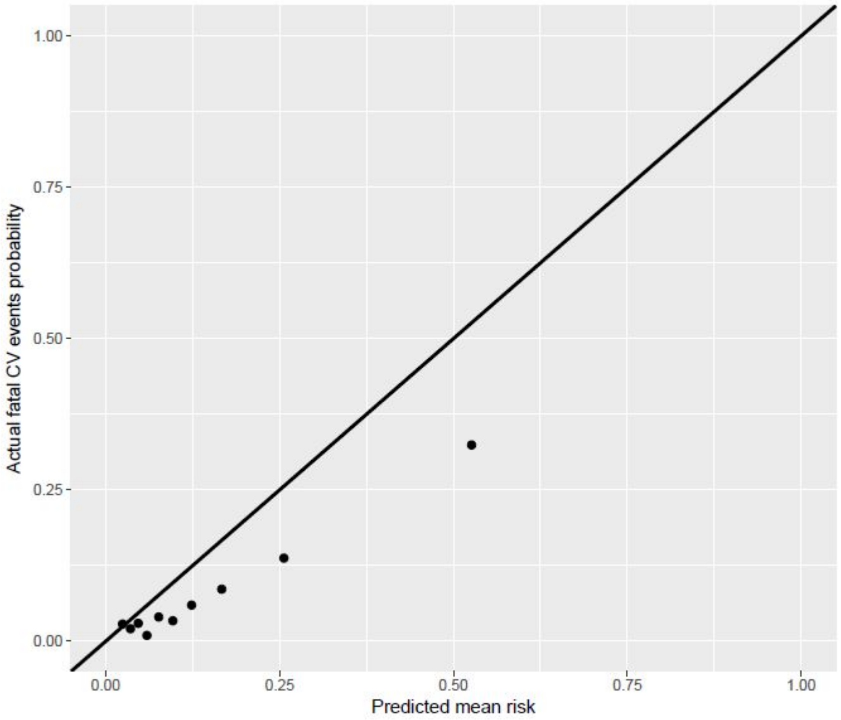

h) SCORE OP-L 5y 'worst-case' scenario for classification of unknown cause of death

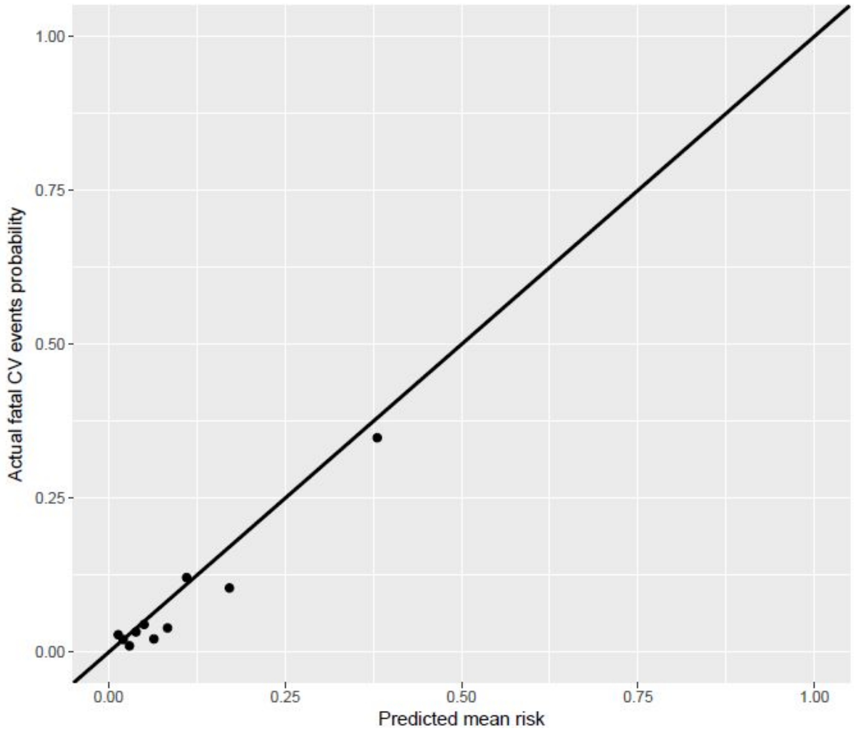

i) SCORE-H 'worst-case' scenario for classification of unknown cause of death

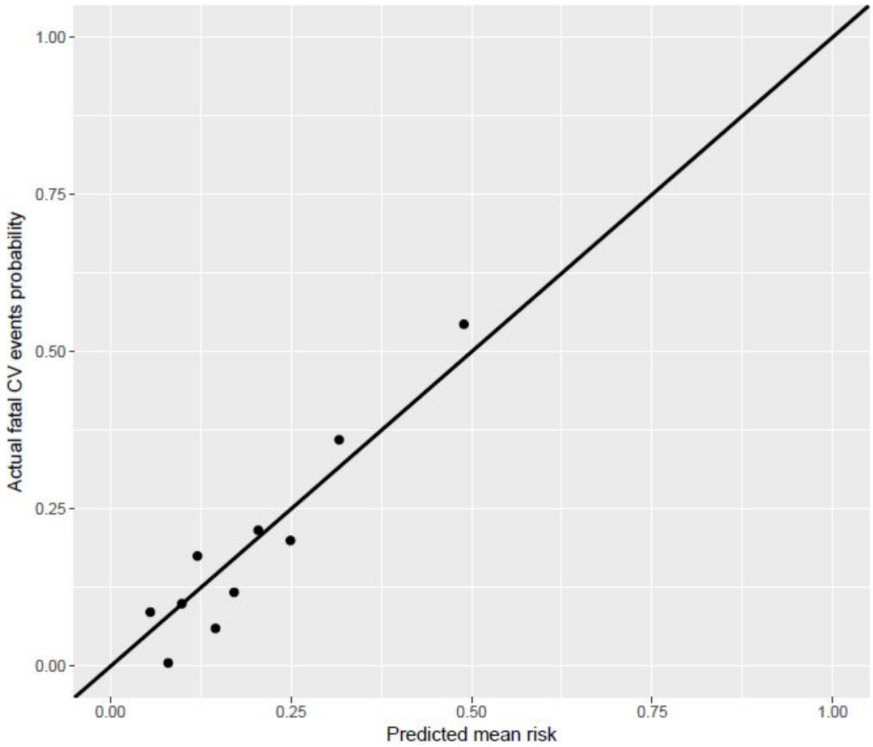

j) SCORE-L 'worst-case' scenario for classification of unknown cause of death

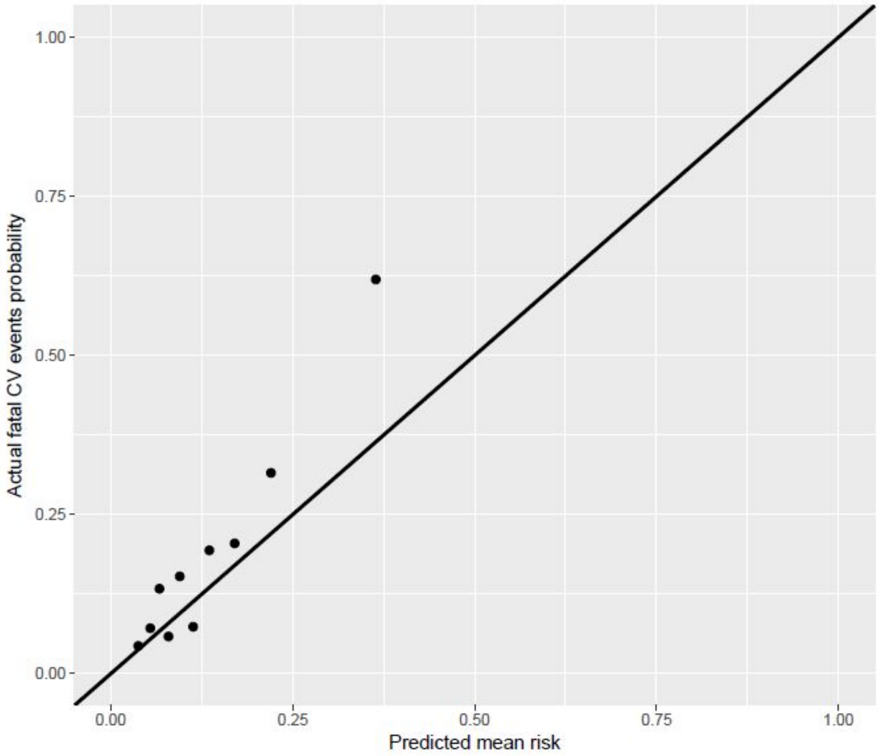

k) SCORE OP-H 'worst-case' scenario for classification of unknown cause of death

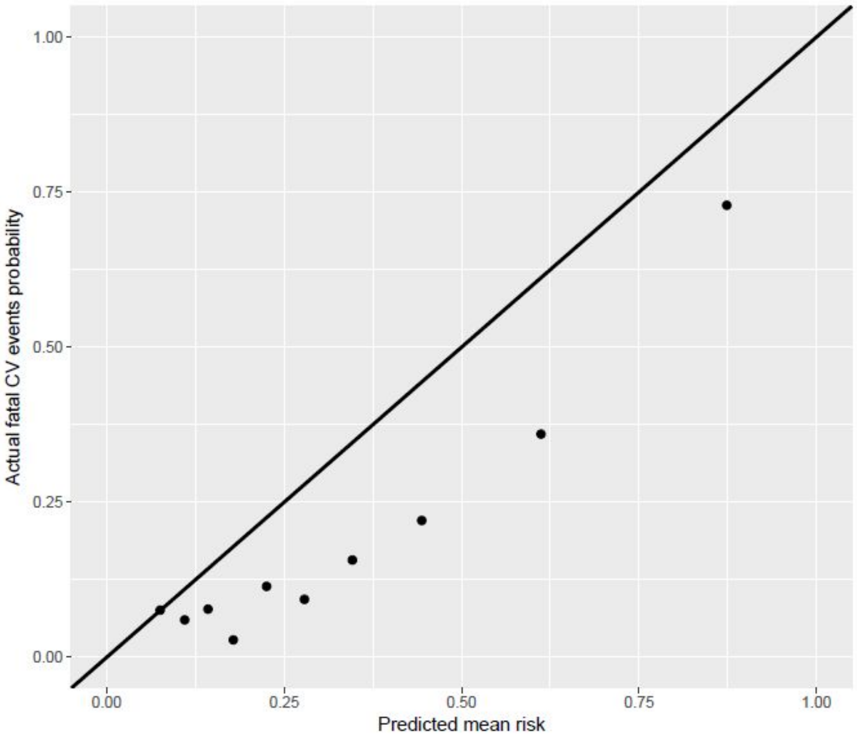

l) SCORE OP-L 'worst-case' scenario for classification of unknown cause of death

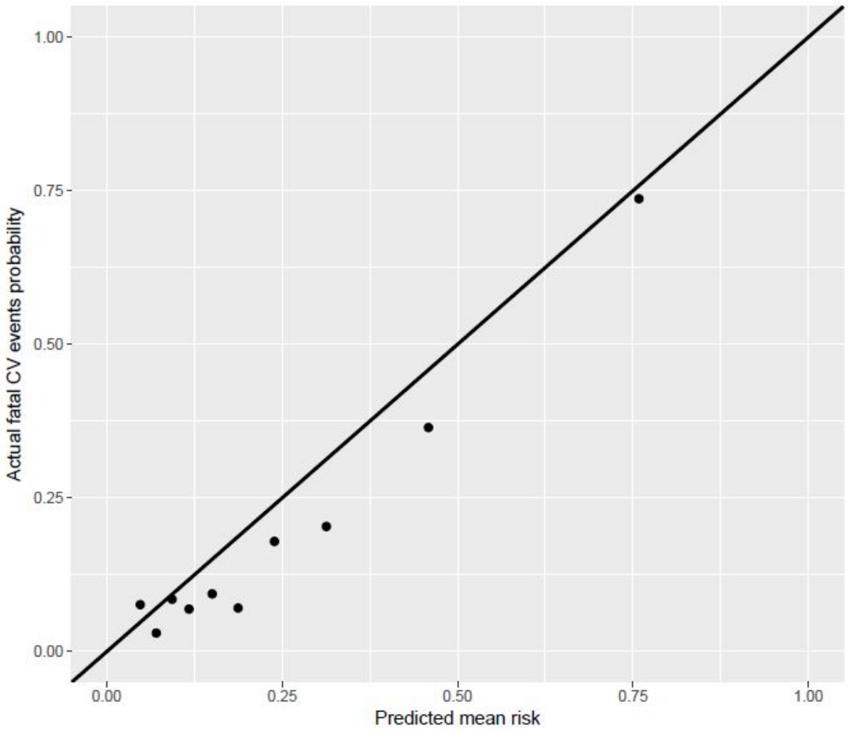

S1 R code

```
# Performance of risk prediction scores for cardiovascular mortality in older persons:
# External validation of the SCORE OP and appraisal

# Risk score function codes:
# Note: the following package must be installed (if not already installed):
# install.packages("SurvRegCensCov")

## SCORE function (modified with mathematical correction)
conroy2003_m<-function(age,sex,high_risk_region,SBP_mmHg,serum_chol_mmol_on_L,smoking) {

a_CHD<-(sex=="M")*(high_risk_region==0)*(-22.1)+(sex=="F")*(high_risk_region==0)*(-29.8)+(sex=="M")*(high_risk_region==
1)*(-21.0)+(sex=="F")*(high_risk_region==1)*(-28.7)

a_no_CHD<-(sex=="M")*(high_risk_region==0)*(-26.7)+(sex=="F")*(high_risk_region==0)*(-31.0)+(sex=="M")*(high_risk_regio
n==1)*(-25.7)+(sex=="F")*(high_risk_region==1)*(-30.0)

p_CHD<-(sex=="M")*(high_risk_region==0)*(4.71)+(sex=="F")*(high_risk_region==0)*(6.36)+(sex=="M")*(high_risk_region==1)
*(4.62)+(sex=="F")*(high_risk_region==1)*(6.23)

p_no_CHD<-(sex=="M")*(high_risk_region==0)*(5.64)+(sex=="F")*(high_risk_region==0)*(6.62)+(sex=="M")*(high_risk_region=
=1)*(5.47)+(sex=="F")*(high_risk_region==1)*(6.42)
  s0_age_CHD<-exp(-exp(a_CHD)*(age-20)^p_CHD)
  s0_age_no_CHD<-exp(-exp(a_no_CHD)*(age-20)^p_no_CHD)
  s0_age10_CHD<-exp(-exp(a_CHD)*(age-10)^p_CHD)
  s0_age10_no_CHD<-exp(-exp(a_no_CHD)*(age-10)^p_no_CHD)
  w_CHD<-0.24*(serum_chol_mmol_on_L-6)+0.018*(SBP_mmHg-120)+0.71*smoking
  w_no_CHD<-0.02*(serum_chol_mmol_on_L-6)+0.022*(SBP_mmHg-120)+0.63*smoking
  s_age_CHD<-s0_age_CHD^exp(w_CHD)
  s_age_no_CHD<-s0_age_no_CHD^exp(w_no_CHD)
  s_age10_CHD<-s0_age10_CHD^exp(w_CHD)
  s_age10_no_CHD<-s0_age10_no_CHD^exp(w_no_CHD)
  risk_CHD<-1-s_age10_CHD/s_age_CHD
  risk_no_CHD<-1-s_age10_no_CHD/s_age_no_CHD
  prob<-1-(1-risk_CHD)*(1-risk_no_CHD)
  return(prob)
}

## SCORE OP function
cooney2015<-function(age,sex,high_risk_region,SBP_mmHg,serum_chol_mmol_on_L,smoking,HDL_chol_mmol_on_L,diabete
s,years) {
  CHD_pred<-(sex=="F")*(
0.121*(serum_chol_mmol_on_L-6)+0.007*(SBP_mmHg-120)+0.147*(age-65)+0.629*smoking-0.559*(HDL_chol_mmol_on_L-1)
+0.832*diabetes)+(sex=="M")*(
0.210*(serum_chol_mmol_on_L-6)+0.008*(SBP_mmHg-120)+0.118*(age-65)+0.584*smoking-0.411*(HDL_chol_mmol_on_L-1)
+0.610*diabetes)
  no_CHD_pred<-(sex=="F")*(
0.007*(SBP_mmHg-120)+0.162*(age-65)+0.428*smoking-0.150*(HDL_chol_mmol_on_L-1)+0.654*diabetes)+(sex=="M")*(
0.007*(SBP_mmHg-120)+0.138*(age-65)+0.567*smoking-0.284*(HDL_chol_mmol_on_L-1)+0.475*diabetes)

CHD_s0<-(years==10)*(sex=="F")*(high_risk_region==1)*0.980247+(years==10)*(sex=="F")*(high_risk_region==0)*0.990066+
(years==5)*(sex=="F")*(high_risk_region==1)*0.99342114+(years==5)*(sex=="F")*(high_risk_region==0)*0.99686523+(years=
=10)*(sex=="M")*(high_risk_region==1)*0.950969+(years==10)*(sex=="M")*(high_risk_region==0)*0.977713+(years==5)*(sex=
="M")*(high_risk_region==1)*0.98487626+(years==5)*(sex=="M")*(high_risk_region==0)*0.99065776

no_CHD_s0<-(years==10)*(sex=="F")*(high_risk_region==1)*0.984806+(years==10)*(sex=="F")*(high_risk_region==0)*0.9888
39+(years==5)*(sex=="F")*(high_risk_region==1)*0.99550004+(years==5)*(sex=="F")*(high_risk_region==0)*0.9971497+(year
```

```
s==10)*(sex=="M")*(high_risk_region==1)*0.98063+(years==10)*(sex=="M")*(high_risk_region==0)*0.980119+(years==5)*(sex
=="M")*(high_risk_region==1)*0.99377834+(years==5)*(sex=="M")*(high_risk_region==0)*0.99379587
  CHD_prob<-1-CHD_s0^exp(CHD_pred)
  no_CHD_prob<-1-no_CHD_s0^exp(no_CHD_pred)
  prob<-1-(1-CHD_prob)*(1-no_CHD_prob)
  return(prob)
}

# Simulation of calibration assessment:

# Note: We have simulated data to allow readers to explore our modeling and
# calibration procedures. See our data sharing statement for information on
# how to access original BIS data.
# (A proposal with primary investigator approval required)

## Simulated data
n<-1000
shape=1.3
scale=15
dataset<-data.frame(time_event=rweibull(n,shape,scale=scale),time_censor=runif(n,min=0,max=8))
dataset$status<-ifelse(dataset$time_event<dataset$time_censor,1,0)
dataset$time<-apply(dataset[,c("time_event","time_censor")],1,min)
dataset$age<-rpois(n,60)
dataset$sex<-sample(c("M","F"),size=n,replace=T)
dataset$serum_chol_mg_on_dL<-rnorm(n,140,50)
dataset$SBP_mmHg<-rnorm(n,130,20)
dataset$smoking<-sample(c(0,1),size=n,replace=T)
dataset$HDL_chol_mg_on_dL<-rnorm(n,45,10)
dataset$diabetes<-sample(c(0,1),size=n,replace=T)
dataset$serum_chol_mmol_on_L<-dataset$serum_chol_mg_on_dL/38.67
dataset$HDL_chol_mmol_on_L<-dataset$HDL_chol_mg_on_dL/38.67
dataset$high_risk_region<-rep(0,n)
dataset$years<-rep(10,n)
dataset$out<-survival::Surv(dataset$time,dataset$status)

## Create risk score variable using SCORE OP-L (2015 Cooney et al)
dataset$pred_risk<-cooney2015(dataset$age,dataset$sex,dataset$high_risk_region,dataset$SBP_mmHg,dataset$serum_chol
_mmol_on_L,dataset$smoking,dataset$HDL_chol_mmol_on_L,dataset$diabetes,dataset$years)

## Create decile groups
dataset$pred_risk_cat<-cut(dataset$pred_risk,breaks=quantile(dataset$pred_risk,probs=seq(0,1,0.1)),include.lowest=T,right=T
)

## Predicted probabilities by decile group
predicted_mean<-aggregate(pred_risk~pred_risk_cat,FUN=mean,data=dataset)[,2]
numerosity<-aggregate(pred_risk~pred_risk_cat,FUN=function (x) sum(!is.na(x)),data=dataset)[,2]

## Model to estimate projected probabilities by decile group
survival::survreg(out~ -1+pred_risk_cat,dist="weibull",data=dataset)->mod
SurvRegCensCov::WeibullDiag(out~pred_risk_cat,data=dataset)
actual<-1-exp(-(10/exp(mod$coefficients))^(1/mod$scale))

## Calibration measures
calibration<-data.frame(predicted_cat=levels(dataset$pred_risk_cat),predicted_mean,actual,numerosity,row.names=NULL)
calibration$predicted_events<-calibration$predicted_mean*calibration$numerosity
calibration$actual_events<-calibration$actual*calibration$numerosity
calibration$Chi<-(calibration$numerosity*(calibration$actual-calibration$predicted_mean)^2)/(calibration$predicted_mean*(1-cal
ibration$predicted_mean))
calibration


## Creation of pseudopopulation dataset bootstrapping by age-sex strata
```

```
set.seed(547658768)
dataset_balanced_temp_list<-list()
for (i in 1:length(summary_berlin_2010$age_cat)) {
dataset_balanced_temp_list[[i]]<-dplyr::sample_n(dataset[dataset$age_cat==summary_berlin_2010$age_cat[i] &
dataset$sex==summary_berlin_2010$sex[i],],summary_berlin_2010$pop2010[i],replace=T)
}
dataset_balanced<-do.call(rbind, dataset_balanced_temp_list)
```

Supplemental References

1.Berlin Brandenburg Amt Für Statistik. Statistisches Informationssystem Berlin Brandenburg (StatIS-BBB) [Internet]. [cited 19 Mar 2019]. Available: www.statistik-berlin-brandenburg.de

2. Conroy RM, Pyörälä K, Fitzgerald AP, Sans S, Menotti A, De Backer G, et al. Estimation of ten-year risk of fatal cardiovascular disease in Europe: the SCORE project. Eur Heart J. 2003;24:987–1003.

3. Cooney MT, Selmer R, Lindman A, Tverdal A, Menotti A, Thomsen T, et al. Cardiovascular risk estimation in older persons: SCORE OP. Eur J Prev Cardiol. SAGE Publications Sage UK: London, England; 2016;23:1093–103.
